# Smartphone use by older adult in the healthy ageing process: a theory based on data *

**DOI:** 10.1590/1518-8345.7252.4383

**Published:** 2024-10-25

**Authors:** Adriana Martins Gallo, Juliane Pagliari Araujo, Wanessa Cristina Baccon, Francielle Renata Danielli Martins Marques, Maria Aparecida Salci, Lígia Carreira

**Affiliations:** ^1^ Universidade Estadual de Maringá, Maringá, PR, Brazil; ^2^ Instituto Federal do Paraná, Astorga, PR, Brazil; ^3^ Scholarship holder at the Coordenação de Aperfeiçoamento de Pessoal de Nível Superior (CAPES), Brazil; ^4^ Universidade Estadual de Londrina, Londrina, PR, Brazil; ^5^ Instituto Federal do Paraná. Londrina, PR, Brazil

**Keywords:** Aged, Healthy Aging, Grounded Theory, Smartphone, Health Promotion, Social Media

## Abstract

to understand the feelings and recognitions of older adults when they experience the use of smartphones in everyday life, as well as the implications for the healthy aging process.

qualitative research, using the Unified Theory of Acceptance and Use of Technology; the Senior Technology Acceptance & Adoption Model and the methodological framework of Constructivist Grounded Theory. We intentionally recruited people aged 60+; smartphone users; participants for 6 months or more in an extension project, without cognitive impairment, determined by theoretical sampling. A semi-structured script guided data collection. Constant and concomitant comparative analysis followed the steps of initial and focused coding, supported by memos, until theoretical saturation. The Sankey diagram was used to analyze the data, using Atlas.ti software.

37 participants were allocated to two sample groups. We present the category “Experiencing technological evolution during the aging process” and two subcategories: “Experiencing feelings when aging in the face of technological development”; “Using the smartphone and recognizing new experiences”.

when experiencing the use of smartphones, older adult understand and recognize the search for digital skills based on their need for usefulness and applicability of information and communication technology in their daily lives, integrating it into the healthy ageing process.

## Highlights

**(1)** Older adult are still segregated from young people when it comes to technology.

**(2)** On a daily basis, older people recognize the need, usefulness and applicability of the smartphone.

**(3)** Older adult are gradually moving towards digital skills.

**(4)** Digital tools are means of enhancing healthy ageing.

**(5)** WhatsApp ^®^ groups help older people to engage virtually with peers.

## Introduction

 The world has experienced two major transitions in recent years: the rapid ageing of the population ^(^ 1 ^)^ , inverting the demographic pyramid and projecting an increase in the number of older adult in relation to young people in the coming decades ^(^ 2 ^-^ 4 ^)^ , and the technological transition, which encompasses the universalization of Information and Communication Technologies (ICT) and has gradually replaced analogue resources and devices with innovative digital models with Internet access ^(^ 5 ^-^ 6 ^)^ . Thus, the experience of technological innovations generates changes in social connections with impacts on health, through the dissemination of information capable of modifying behaviors that affect social isolation, loneliness and communication, especially in older people ^(^ 7 ^)^ . The use of smartphones by the older adult in their daily lives is driven by meanings that strengthen social bonds, feelings and recognition of experiences in pairs in a technologically connected context ^(^ 8 ^)^ . 

 Recently, there has been scientific interest in the potential of ICT to support an inclusive, healthy and active ageing process ^(^ 9 ^-^ 13 ^)^ and aligned with the proposals of the Decade of Healthy Ageing (2021-2030) and the Sustainable Development Goals (SDGs) ^(^ 2 ^-^ 4 ^,^ 11 ^)^ , affirming digital technology as a transformative agent capable of boosting the independence and productivity of older people ^(^ 1 ^)^ . 

 As an important form of communication, smartphones, their applications and social networks are present in people’s lives ^(^ 13 ^)^ and in Brazil, in 2023, the ownership of this equipment in households (95%) surpassed, in all regions, traditional television (94%) and landlines (12%) ^(^ 14 ^)^ . With the device, 75% of US seniors aged 65 access the web ^(^ 15 ^)^ . 

 It is a fact that there is a disparity between owning the equipment and functionally accessing the Internet. Various conceptual and theoretical models have long been studied ^(^ 16 ^-^ 17 ^)^ , and applied in relation to the acceptability and applicability of technology in the daily lives of older adult. The aim is to contribute to the medium- and long-term impact of this on ageing ^(^ 1 ^,^ 17 ^-^ 18 ^)^ . Different contexts are considered for narrowing the gap between the functional applicability of ICT and just carrying devices as tools. Among them, cultural factors, feelings, personal experiences and social motivations are pointed out as predictors of access ^(^ 19 ^)^ . 

 Digital technologies have replaced and complemented analog ones, reflecting generational changes in communication models over the years ^(^ 5 ^,^ 20 ^)^ . In recent years, ICTs have been used as strategies in family systems to promote communication, avoid social disruption, have a positive impact and develop new care roles among family members ^(^ 21 ^)^ . 

 It should be noted that social isolation is an unfavorable condition that impoverishes bonds and marginalizes the older adult, and that most of them like to get involved in social activities that bring meaning to their lives ^(^ 7 ^,^ 22 ^)^ . By allowing themselves to experience the networked world, older people can take control of their lives via the Internet and feel part of a community ^(^ 23 ^)^ . 

 Studies that analyzed device properties and the use of ICT by older people found that Internet access was more frequent by older people using smartphones than those using tablets and computers ^(^ 11 ^,^ 24 ^)^ and that social connection can be promoted with this tool, offered in a safe and viable way ^(^ 22 ^)^ . 

 By understanding the urgency of the fight against ethicism - changing the way we think (stereotypes), the way we feel (prejudices) and the way we act (discrimination) ^(^ 4 ^)^ - the need arises to give visibility to the ageing person who is digitally engaged and needs support, including professional support, by understanding their feelings and anxieties. 

 Countries such as China are already studying the need to encourage nursing to reduce the digital divide by increasing digital skills among older people ^(^ 25 ^)^ . The Brazilian context still lacks research that reinforces the alignment of the proposal to use ICT to optimize nursing education strategies for the older adult. 

To this end, it is extremely important to know how the protagonists adopt technological resources as they get older and apply them throughout their lives. The question then arises: what do older adult who are getting older feel and experience when they habitually use the resources of a smartphone? The aim is to understand the feelings and recognitions of older adult when they experience the use of smartphones in their daily lives, as well as the implications for the healthy ageing process.

## Method

### Study design

 Qualitative research, aligned with the conceptual framework of the Decade of Healthy Ageing 2021-2030 baseline report ^(^ 2 ^,^ 4 ^)^ using the Unified Theory of Acceptance and Use of Technology (UTAUT) as a theoretical model. ^(^ 16 ^)^ and the Senior Technology Acceptance and Adoption Model (STAM) ^(^ 17 ^)^ . The methodological orientation that underpinned the study was Data-Driven Theory (DDT), from the Constructivist perspective ^(^ 26 ^)^ . Methodological rigor was outlined using the Consolidated Criteria for Reporting Qualitative Research (COREQ) checklist ^(^ 27 ^)^ . 

### Study setting and selection of participants

 The setting was a community outreach project ^(^ 18 ^)^ , linked to a federal public educational institution and which has a partnership with a municipal center for the older adult located in the southern region of Brazil. The project aims to promote digital and health skills, such as the use of smartphones by older people, in a way that is guided by professionals, as a resource for providing health and well-being and integrating the guidelines that promote healthy ageing ^(^ 19 ^)^ . 

 Potential participants were intentionally selected based on a set of inclusion criteria: a) people aged 60 or over; b) smartphone users with Internet access; c) participants in the “ *Vovôs e Vovós Conectados* ” extension project for at least 6 months. ^(^ 18 ^)^ , d) and without cognitive impairment. The Mini Mental State Examination (MMSE) was used to assess the latter criteria. Older adult with a score of 27 or more points were included ^(^ 28 ^)^ . There were no exclusion criteria. 

**Figure 1:**
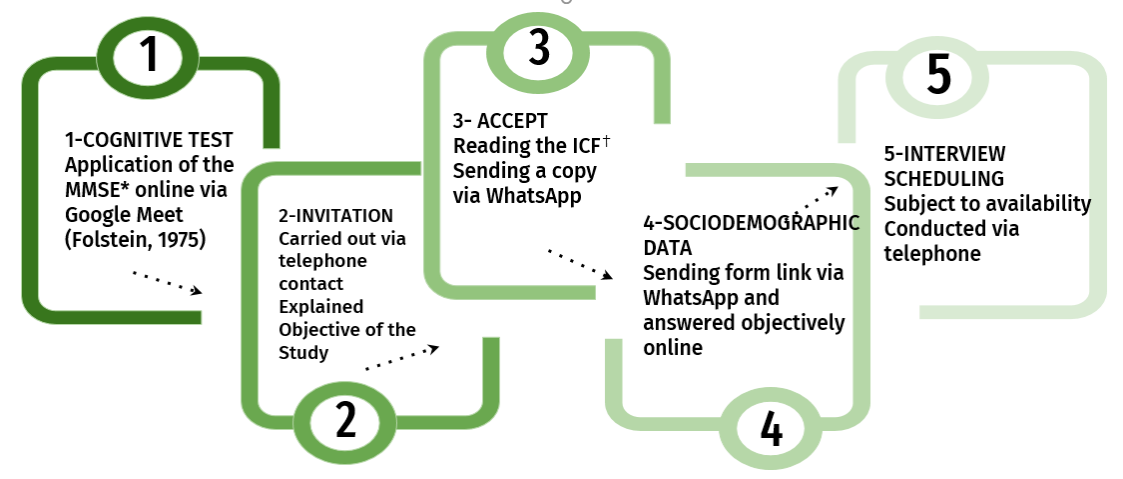
- Steps of the participant selection process (n = 37). Maringá, PR, Brazil, 2023

 A theoretical sampling approach was used, an assumption of the CGT ^(^ 26 ^,^ 29 ^)^ .The study was designed to include a diverse group of older adult with a variety of characteristics and experiences. Initially, the lead researcher established a relationship with the potential participants and then proceeded with the other stages ( Figure 1 ). 

 *Mini Mental State Examination; ^†^ Free and Informed Consent Form 

Participants were recruited regardless of gender, race or family support, allowing for maximum variation in participant characteristics. As a result, 37 people took part in the study and were organized into two sample groups, with the first sample group comprising 27 older adult with family support and the second group including 10 older adult without family support, who lived alone in their homes.

 The achievement of theoretical saturation was signified from the moment that no additional empirical data was generated during the conceptual investigation phase, thus the theoretical sampling was determined with the number of participants ^(^ 26 ^)^ . Family support improves physical and mental health-related quality of life in older people ^(^ 30 ^)^ and life satisfaction ^(^ 31 ^)^ . Thus, significant and positive relationships between social support and smartphone use have already been shown in the literature ^(^ 32 ^)^ provided the justification for creating both sample groups. Sample size guidelines recommend a range of 20 to 30 participants to reach saturation in a data-driven study ^(^ 33 ^-^ 34 ^)^ . Although our sample consisted of 37 people in total, by the 28th interview the central category had already been identified and dimensionalized. 

### Data collection procedure

 Data collection took place from August 2022 to March 2023 using a regular phone call or teleconference (meet), or video via the WhatsApp ^®^ app, and only the researcher and the participant were present for the call. 

The Informed Consent Form (ICF) and the image and voice authorization, as well as verbal consent, were signed and made available digitally to the participant.

 The individual interviews were guided by a guide drawn up by researchers from the research group NEAPE - *Núcleo de Estudos Avançados e Pesquisas sobre Envelhecimento* (Center for Advanced Studies and Research on Aging) -, who contributed their expertise in the area and, based on the literature, their experience in the field ^(^ 4 ^,^ 16 ^-^ 17 ^,^ 35 ^)^ . The guide focused on the following areas: understanding older people’s experience of using the Internet via smartphone; its perceived usefulness; facilitators, feelings and constraints when using smartphones; and the implications of technology for older people’s health and well-being. 

The application of the guide in the first two semi-structured interviews was evaluated for comprehensibility, comprehensiveness and clear and concise language, and the questions were adapted as the collection progressed. The two interviews were integrated into the study and there was no need to repeat either of them.

Data collection was carried out by the main researcher, who has received training in simultaneous data collection and analysis, has experience in qualitative research and with older adult who use virtual social networks, and has a dual background in nursing and ICT management, favoring a differentiated and welcoming interaction at the time of the interview.

 From the inferences made during the data collection process and the concomitant analyses, issues related to family support in the older adult person’s home emerged, which were noted and recorded in memos, as presupposed by the CGT ^(^ 26 ^)^ , two different sample groups with these characteristics were organized. 

Older adults who live unaccompanied were included in the study, as this was a different context from the first group, in which the older adult person sharing a home is more likely to be in an environment with resources, tools and people familiar with digital technologies, especially the smartphone.

 The collection was closed due to theoretical saturation, which is the point at which no insight into new concepts is generated and so evidence was produced repeatedly to conceptualize the main category ^(^ 29 ^)^ . 

 The semi-structured interviews were recorded with the consent of the participants, and were de-identified, transcribed verbatim, using the Reshape ^®^ resource to help maintain their completeness, and analyzed. The data collection and analysis process took place simultaneously. 

The duration of the interviews varied between 31 and 72 minutes and lasted an average of 40 minutes. The researcher’s reflections were recorded in the form of memos, which were written up throughout the process as the collection progressed, and were fundamental in supporting the integration, the reporting of conceptual schemes, illustrating the development of ideas and codes, guiding the construction of theory.

 There was no need to return the documents to the participants for possible corrections and adjustments. After the documentation had been rigorously checked, the material was exported to Atlas.ti ^®^ software. The quotes from the participants were identified: numbered sequentially; F or M (female or male), followed by the characterization of housing (alone or accompanied); age in years. 

### Data analysis

 Data coding according to the Constructivist CGT ^(^ 26 ^)^ , was developed in two stages, initial and focused. In the first, each action from the data was explored and coded incident by incident in order to understand the information based on the meanings and experiences of the participants, forming the first dimensions of the analysis of the experience. 

In the second, focused stage, the main, more rigorous analysis took place, enabling an analytical understanding of the phenomenon by promoting conceptual groupings until categories and subcategories were identified.

Throughout the process, the data is constantly compared and, in this method, the researcher moves between inductive and deductive thinking, generating a relationship between the categories and articulating the CGT between interpretation and integration of new data.

The Sankey diagram, used here to represent data integration, is a classic figure that shows the energy efficiency of data flow and connections. If nodes have incoming and outgoing links in the currently visible set of nodes, they are placed in intermediate bands or layers, showing the strength of co-occurrence between pairs of nodes. Simply put, the bands on either side of the diagram represent conceptual subcategories relating to the data analyzed and named, and their width indicates the energy efficiency of the data flow (substantial qualitative and quantitative relationships between the subcategories crossed represented by the participants’ statements).

The wider the band, the more representation there is of the data flow or the intensity of the substantial relationships and, as the initial color of the band changes, it signifies crossing with other bands, representing interaction between data flows from other subcategories, i.e. crossings between themes.

### Methodological rigor

 The criteria of credibility, originality, resonance and usefulness conferred the rigor of the research and the validity of the CGT ^(^ 26 ^)^ . Credibility was guaranteed by the detailed care taken with the data during the transcription of the interviews; in the construction of the memos, which were constantly compared and contrasted, as well as the researcher’s prolonged involvement in order to gain a deep understanding of the phenomenon being studied. The bond with each participant fostered a harmonious and trusting relationship, favoring the sharing of experiences. Another strategy to increase credibility was to reach data saturation, when the study participants no longer provided new information ^(^ 36 ^)^ . 

 To facilitate discussion among peers, originality was evidenced through memos and schematic representations of the phenomenon associated with the researcher’s reflective process ^(^ 37 ^)^ . A combination of deductive and inductive analysis, fostered by the CGT method, was used to ensure resonance and create concepts that accurately reflect participants’ experiences. 

Finally, usefulness encompasses the ability to deepen research participants’ understanding of their daily experiences, the establishment of a basis for policy development and good practice.

### Ethical aspects

The project was approved by the National Research Ethics Committee, under opinion no. 5.553.406 and CAAE: 57993522.0.0000.0104. The ethical precepts established by Resolutions 466/2012 and 510/2016 of the National Health Council were observed.

## Results

**Figure 2:**
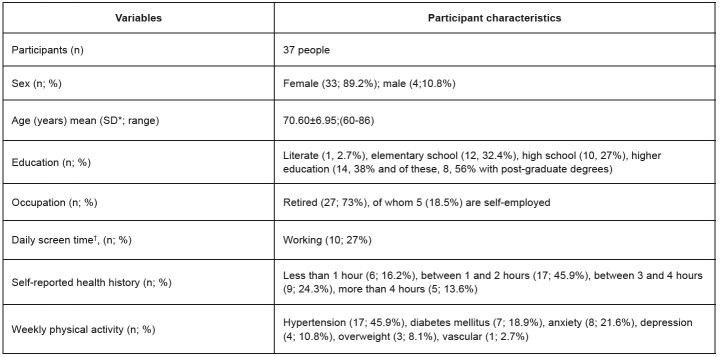
- Characterization of study participants (n = 37). Maringá, PR, Brazil, 2023

 Thirty-seven older adult took part in the study ( Figure 2 ). Most of them have been smartphone users for at least 24 months and access it for more than an hour a day, showing considerable familiarity with the technology offered by the device. 

**Figure 3:**
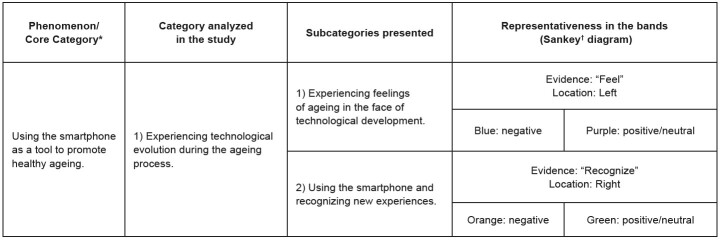
- Relationship between Data-Based Theory, Categories and Subcategories/Sankey Diagram. Maringá, PR, Brazil, 2023

 In order to arrive at the phenomenon, interactions and relationships between the categories and subcategories permeated the entire data set, unifying the various perspectives and aspects observed, providing a comprehensive and complete understanding. The data includes the central category and its two subcategories ( Figure 3 ). 

*Standard deviation; ^†^ Self-reported screen time, referring to smartphone use in a 24-hour period

**Figure 4:**
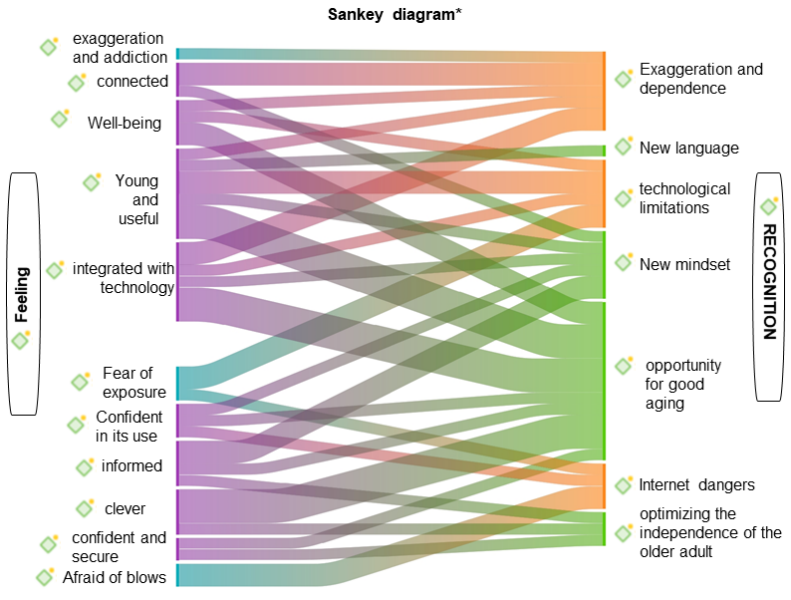
- Flow integration of subcategories “Experiencing feelings when aging in the face of technological development - Feelings” and “Using the smartphone and recognizing new experiences - Recognitions” of the participants (n = 37). Maringá, PR, Brazil, 2023

*Phenomenon/Core Category referring to CGT (Doctoral Thesis); ^†^ Sankey diagram ( Figure 4 )

Older adult’s experiences of using a smartphone and how to recognize these experiences are presented in two subcategories: “Experiencing feelings of ageing in the face of technological development” and “Using a smartphone and recognizing new experiences”. For a broad visualization and better understanding of the co-occurrences, representative verbs were grouped/standardized in each subcategory, which co-occurred with each other, with subcategory 1 being the verb “to feel” and subcategory 2, the verb “to recognize”, representing the experiences and the use of the smartphone, respectively, within the context and objective of the study.

 In view of the codes analyzed, with the support of Atlas.ti ^®^ software resources, it was possible to identify in each category the positive/neutral and negative reactions in relation to the theme, and the coding was done by color identifying each of these reactions. The greatest interactions identified by crossing the explicit bands in the Sankey diagram ( Figure 4 ) were evident in the interaction of recognizing opportunities for ageing and the relationship with opportunities for integration in the technological context, with confidence in the search for information. 

 *Sankey diagram created using Atlas.ti ^®^ software 

Both categories, represented in the diagram, express the experiences of the participants, in each of the interceptions and their representations as they unfold throughout their experiences, reflecting on the aging process. After the smartphone was introduced into the daily lives of these older adult, they changed some ways of thinking and acting in relation to various situations, including the fluidity with which information arrives and keeps them up to date with various contexts, from the local to the global.

 Taking part in this process is an opportunity that, for many, generates recognition and feelings that place them in a social and individual context, instilling ideas that resonate in the long term, including the adoption of healthy habits. *[...] we get to know more about the things we want to know and we get to know them right away, it’s very good* (2-F-Alone, 67) *. [...] wherever I am, my cell phone is with me, otherwise I’d have to wait until I got home to call someone or go to people’s houses. Not now, with my cell phone in my hand I can communicate, look up things I need, do everything* (26-F-Accompanied, 72) *. I use my cell phone to remember basic things, like water. Right now I have my bottle next to me. I’ve had chronic urine infections, I didn’t used to drink water* (30-F-Accompanied, 70). 

 By connecting to networks, perceiving themselves to be more informed and able to search for information, older people recognize themselves as more independent and with a mindset adjusted to contemporary aspects of technology, even if they declare the need for continuous learning. In their feelings, they explain their understanding of what is real and what is virtual. *[...] when I worked, I didn’t have the Internet, but I did have a fax machine [...] we live according to what’s evolving in the world, if something else comes along in the future besides the Internet, it’ll stay in the past and I’ll adapt to the new* (11-F-Single, 68) *. [...] it’s not difficult for me, I don’t see any major difficulties. Some that I have, if I go after it, I learn* (28-F-Self, 67). 

From the perspective of participating, reconciling and alternating between the real and the virtual, older people seek support in the virtual mode, through their smartphone, connecting with people, places, services and information, recognizing this mode as a tool that can offer a range of opportunities, including in the aging process.

 By understanding the virtual environment as a graphic representation, something transmuted and applied to real situations, older people project the future with feelings of optimism based on the influences they receive on the web. *[...] when I go out, I see the address and location on the virtual map. It’s made it easier for me because I’m terrible at remembering street names, so this cell phone location helps me a lot* (10-F-Accompanied, 65) *. [Look how cool it is, we’re talking and planning (online) in a group with four childhood friends, one of whom is from outside Brazil, and we’re going to save up for a cruise in two years’ time. So that’s growing old wisely and healthily. It’s looking to the future in two years’ time, I’ll be traveling with my friends, it’s an optimism I haven’t had for a long time* (20-F-Alone, 60) *.*

 People perceive the possibility of ageing connected to a network as an opportunity and a privilege compared to those who don’t have the same access. *I wish all people our age had this possibility, or interest in finding out about their health, because the possibilities today on the Internet are not as difficult as they used to be. If you have this interest... older people can do it.* (30-F-Accompanied, 70) *. [...] I have the opportunity to exercise my mind, and age better* (32-F-Accompanied, 60). 

 Fragmentation from the perspective of two worlds, real and virtual, complement each other. Although the fight against ageism is a challenge of the decade, older adult themselves carry stigmas linked to the entitlement of feeling like they belong to two realities, referring to the first, a more youthful and socially accepted reality due to the chance to participate in this technological context and the second, a segment of retired people, but inserted and engaged in the opportunities offered by technology. *[...] as for technology, I can only say that it has been great for me. Growing old in a world of modernism is a great opportunity for our generation* (22-F-Accompanied, 63). *Using the Internet and cell phones in this world full of technology is very important to me, because I see several opportunities to grow through this means* (37-M-Accompanied, 73). *[...] I couldn’t stay at home when I retired, it was all of a sudden, and the cell phone helped me a lot. I would read about various subjects and that helped me become who I am today, more youthful, because after 60 it is complicated, it is difficult to look in the mirror and see that you have reached an age where people do not accept you everywhere with the same attention as before* (20-F-Alone, 60). 

 The feeling of well-being is perceived in light of the satisfaction of understanding aging as an irreversible process, but one that can be constructed in a healthy way by taking care of one’s health in a global way. *No matter how much I participate in physical activities at the community center to prepare myself to age well, there will come a time when I will be more limited. I feel good using my cell phone during this time, knowing that I have the capacity to participate and learn, both intellectually and physically* (22-F-Accompanied, 63). *I was in a very deep depression, almost reaching physical violence, in this sense the group helped me a lot with the social part, starting to talk, listen, read, videos of old objects that I can revisit, it seems to rescue our soul, it goes to the bottom of the well and pulls us out and says... look at all this history you have in your life, it was very pleasant and has been very good! It changed my life* (20-F-Alone, 60). 

 On the other hand, they feel concerned about specific situations, such as scams in the virtual environment that are common and victimize older adult. The fear of excessive exposure on virtual social networks is a feeling that accompanies older adult connected to the Internet due to the publication of personal data. *[…] the person doesn’t know that the message is malicious and passes it on to us [...] there are also people who have ulterior motives, and by passing it on to a third party, they are committing a crime* (8-M-Accompanied, 86) *. I feel apprehensive and wary of money and banking matters, I don’t do anything about that* (12-M-Accompanied, 86). *[...] it’s not a lack of guidance (falling for the scam), but it’s a minute of foolishness and that’s it* (22-F-Accompanied, 63) *. [...] I’m afraid because there (on the network) we have almost no privacy, I don’t post photos, I don’t comment on my comings and goings, I’m very exposed. I don’t participate in social networks, I don’t like all that exposure, I prefer more discretion* (10-F-Accompanied, 65). 

When they fear dangers on the Internet, they recognize and attribute weaknesses related to the limitation of technological knowledge, differing from young people who are already digital natives and have aptitude in this area inherent to human development.

 Older adult, even with limitations, recognize that the new way of thinking, with the use of smartphones, allows participation and engagement in what they call the “rhythm of technology”. *[...] A child picks up a cell phone and messes with everything, we have more difficulty because we started very late* (1-F-Accompanied, 70). *[...] the older adult are very important in society, despite everything, we see so much prejudice... but there is still a target, a look at this older adult public that is also making the machine go round. I feel “in the rhythm” being part of the technological process... Before, at 50 years old, I was already an old and silly person, I was good for taking care of grandchildren, not today, it is very different and I think it is commendable, great* (22-F-Accompanied, 63). *Today, when I opened the document we read (TCLE) I clicked on the pen, and the signature I made appeared, [...] I felt smart, I remembered how to do it and I did it right away* (28-F-Alone, 67) *. I feel like I am keeping up with the pace of technology because I learn a lot and even teach. There are things I learned that my grandchildren didn’t know, like the black microphone, press to speak. They didn’t know that, and I taught them* (29-F-Alone, 69). 

## Discussion

This study understood what older adult feel and recognize when using smartphones in their daily lives and how this experience affects the healthy aging process. It differs from previous studies that approach older adult as technology learners and innovates by focusing on them as protagonists, users of technological resources, and endowed with a new mindset in search of digital skills to apply throughout their lives.

 We corroborate previous studies ^(^ 9 ^,^ 35 ^)^ , when we point out that older adult, when perceiving smartphones as a benefit in daily life, have been using them more and more in their routines and this has become a determining factor for their inclusion in various personal activities. However, the willingness of older adult to use technologies varies according to their expectation of benefit and the belief that they help them stay safe ^(^ 16 ^-^ 17 ^,^ 35 ^,^ 38 ^)^ . 

 We present evidence that shows that older adult feel more intellectually independent and autonomous in their daily activities when using smartphones. Another study notes that most theories of aging do not consider the individual, social or cultural variability of older adult’s goals and expectations ^(^ 38 ^)^ . 

 In the daily use or applicability of technological advances in the lives of older adult, independence is perceived as an important factor for successful aging ^(^ 38 ^)^ . However, we present, in an individualized context, the recognitions and feelings of the technologically engaged older adult person, and from this, corroborating our results, we understand that in order to build a meaningful life for the older adult, it is necessary to belong, recognize, and feel. Thus, our results pointed to the interaction of several feelings from experiences with the use of smartphone technology, and the cross-occurrence between the recognition of these feelings and their representations in the unfolding throughout their experiences, provide an opportunity for healthy aging as proposed by the Sankey diagram. 

 Regarding the perceived usefulness of the technology acceptance point proposed by the UTAUT model ^(^ 16 ^)^ and STAM theory ^(^ 17 ^)^ , Older adult need to consider the applicability of technology and its tools in their daily lives. This study clearly shows that, even if they use their smartphones functionally and this satisfies them, they need to be involved in the dynamics of continuous learning. The experiences that underpin the feelings of older adult when using smartphones are related to their own perception of competence and confidence in handling the equipment ^(^ 22 ^)^ . 

 In China, a study with older adult on reasons for using smartphones showed that communication and the search for information influence life satisfaction, and this was fully mediated by digital literacy ^(^ 39 ^)^ , reaffirmed by our results. 

 Therefore, it is important to understand that social factors, such as family and peer support and cultural patterns, help older people acquire digital skills, and their use in everyday life reflects changes that promote healthy aging ^(^ 1 ^)^ . 

 In addition to informal support, professionals from a variety of areas can help older people, especially health professionals. Exacerbating barriers to accessing in-person care, they can recommend content and apps that contain health information ^(^ 25 ^)^ . In the UK, more than half of older people aged 65 and over are willing to use digital health resources. However, the willingness to use health apps recommended by professionals decreases as age increases ^(^ 12 ^)^ . These data reinforce the urgency of addressing issues related to this topic with older people, understanding their needs. 

 In China, there is a discussion about the priority that should be given to removing some obstacles in the provision of tools, including product design, which focuses exclusively on young people and their priorities, age-related prejudice, and an external environment that is anti-facilitative and inductive. In other words, technologies are targeted according to age and infrastructure, but they must be rethought according to the needs of the aging process ^(^ 40 ^)^ . 

 Some findings show that there is no unanimity about the benefits or harms experienced by older adult through the adoption of the recurrent practice of smartphone use and thus there is a fusion for the perceived/recognized influences ^(^ 3 ^)^ . This study showed a feeling of optimism, reflected in feelings of perceived well-being, which was demonstrated in the Sankey diagram in co-occurrence with other feelings. Other studies report positive issues about the influences that older adult receive when using smartphones and applying them in their lives ^(^ 3 ^,^ 41 ^-^ 42 ^)^ . 

 The different feelings experienced by older adult in relation to the opportunity to be connected in a network with other people of the same age group and with the digital world generate experiences of social digital participation, technical competence, improvement of physical and cognitive function ^(^ 22 ^)^ and connection with family members, including in vulnerable situations ^(^ 21 ^)^ . We also provided evidence that older people feel privileged compared to their peers who have not yet advanced in developing skills with digital tools, confirming the results of another study that supports that technology training has a positive impact on smartphone use when it comes to well-being ^(^ 35 ^)^ . 

 The fact of feeling privileged does not eliminate the need to critically examine the way in which others who are under a preconception of marginalization have access. The results of a recent review study that addresses age-related bias in artificial intelligence systems stand out, covering the paths of technological development and reiterating the existence of segregation ^(^ 43 ^)^ . 

 The use of smartphones promotes a connection that transcends age issues, providing older adult with the experience of belonging to the digital world by actively participating in ICT ^(^ 38 ^)^ . 

This study is in line with the conceptual framework of the Decade of Healthy Aging, which focuses on the fight against ageism. According to the Pan American Health Organization, ageism is a form of age discrimination that results in poorer health, social isolation, premature deaths, and generates billions in costs to public coffers globally.

 We highlighted the thoughts of the older adult participants themselves, who recognize some factors that condition or restrict digital competence in relation to young people who are native to this time. Throughout the world, for example in Asian countries such as Japan, Singapore and Thailand ^(^ 1 ^)^ , North America ^(^ 15 ^)^ , and South America, such as Brazil ^(^ 14 ^)^ , there are divergences in relation to the adoption of technology through smartphones between young people and older adults, even though a reduction in the gap between the age groups has been perceived over the decades. 

 Our study suggests that older people have feelings of belonging and integration linked to innovative concepts as they emerge and develop. Thus, stigmas and embarrassments brought on by people themselves contribute to active and successful aging, including ageism in relation to the use and design of digital technology ^(^ 38 ^)^ . In this sense, products developed especially “for” and “by” older people promote the reduction of ageism. 

 Fears related to insecurity, uncertainty and concerns are common among older people who use digital tools for communication. As a negative contribution, we highlight that virtual scams and the fear of excessive exposure can lead older people to withdraw from virtual social networks, becoming a factor that prevents active participation ^(^ 42 ^,^ 44 ^)^ . 

 There is a sense of fear involved in recognizing the presence of a new communication context, with natural limitations of the transition process. Corroborating this, some barriers hinder the use of smartphone technology by older people, including fear, lack of knowledge, concern about complexity, physical ability, and difficulty in communicating ^(^ 35 ^)^ . It should be noted that virtual applications should not be developed specifically for specific audiences, such as the older adult, as this results in ease of adaptation and learning, which, consequently, implies the continued use of virtual tools and devices in the medium and long term ^(^ 9 ^,^ 45 ^)^ . 

 The findings point to weaknesses that are related to the restriction of technological knowledge. Even with these restrictions, feelings of technological engagement were observed among the older adult, reinforcing the results of other studies ^(^ 35 ^,^ 38 ^)^ , which point out that some factors are facilitators and others hinder the use of forms that diversify institutional and family support for guiding personal development with new technologies, in addition to motivations and experiences in the face of the aging process ^(^ 38 ^)^ . 

 We studied the perception and feelings of healthy older adult, however, the literature provides data on associations between the unregulated and excessive use of technology, especially smartphones, by older adult, which impacts sleep quality due to stress and anxiety factors ^(^ 46 ^)^ . 

 Above all, and intentionally, the development of digital skills and technological training for the older adult public must originate from intentions and usage behaviors. This will enable the development of acceptance models in future studies ^(^ 35 ^)^ . 

We identified limitations in this study related to the targeting of evidence only to older adult who already have contact with ICT, incorporating smartphones into their routines and also, guided by professionals for such use, in an extension project. The intentionality of those older adult who still wish to integrate, but who have not had the opportunity, was not considered. We suggest exploring this theme in new investigations and more diverse perspectives in ethnic terms, in a community-based approach, for example, which could lead to a better understanding of the impact of ICT on healthy aging.

From both the perspective of digital social inclusion and from the perspective of health promotion, this article is innovative in that it brings the perception of the effects that the use of technology has on the lives of older adult based on their life experiences and personal motivations, providing support for the development of strategies that can be guided by health professionals and proposals for the community and the older adult population. Aligning the search for digital skills with everyday life helps combat ageism and promote healthy aging, contributing in the long term to the advancement of scientific knowledge in the health and nursing fields.

## Conclusion

Understanding the feelings and recognition of older adult regarding the use of smartphone technology in their daily lives allows us to see that, at times, older adult still live segregated from young people in the context of technology. Even though they feel integrated, recognizing a new mentality and opportunity to optimize independence when using smartphones, the experiences of older adult reveal a fragmentation between real and virtual. However, this fact is not a determining factor for this process to be static. Gradually, through the recognition of their needs in relation to technology, especially ICT, perception of its usefulness and applicability in daily life, identification with peers, the search for digital skills has made older adult protagonists of learning, reverberating throughout their aging.

The permanence of older adult in virtual engagement highlights favorable conditions for promoting health in the aging process. Owning a smartphone does not guarantee its use, nor digital social inclusion, but professional encouragement in virtual groups and guidance on healthy practices integrate them into an environment of resources and information applicable to the healthy aging process.

Additionally, nursing is able to contribute to the decision-making process in the virtual field, and can stand out as a leader, conducting strategies that change the conceptual and practical reality of ageism.
