# Supplementary material for: Smartphone use by older adult in the healthy ageing process: a theory based on data
Source: Rev Lat Am Enfermagem. 2024 Oct 25;32:e4383. doi: 10.1590/1518-8345.7252.4383 (PMC11526248; doi:10.1590/1518-8345.7252.4383)
Supplement: Translation

# Translation: Uso de *smartphone* por personas mayores en el proceso de envejecimiento saludable: una teoría fundamentada en los datos *

**DOI:** 10.1590/1518-8345.7252.4382

**Published:** 2024-10-10

**Authors:** Adriana Martins Gallo, Juliane Pagliari Araujo, Wanessa Cristina Baccon, Francielle Renata Danielli Martins Marques, Maria Aparecida Salci, Lígia Carreira

**Affiliations:** ^1^ Universidade Estadual de Maringá, Maringá, PR, Brasil; ^2^ Instituto Federal do Paraná, Astorga, PR, Brasil; ^3^ Becaria de la Coordenação de Aperfeiçoamento de Pessoal de Nível Superior (CAPES), Brasil; ^4^ Universidade Estadual de Londrina, Londrina, PR, Brasil; ^5^ Instituto Federal do Paraná. Londrina, PR, Brasil

**Keywords:** Anciano, Envejecimiento Saludable, Teoría Fundamentada, Teléfono Inteligente, Promoción de La Salud, Medios de Comunicación Sociales

## Abstract

comprender los sentimientos y reconocimientos de personas mayores al experimentar el uso del *smartphone* en el día a día, así como las implicaciones en el proceso de envejecimiento saludable.

investigación cualitativa, utilizando *Unified Theory of Acceptance and Use of Technology* ; *Senior Technology Acceptance & Adoption Model* y el referencial metodológico Teoría Fundamentada en los Datos Constructivista. Se reclutaron intencionalmente personas de 60 años o más; usuarios de *smartphones* ; participantes desde hace 6 meses o más de un proyecto de extensión, sin deterioro cognitivo, determinados por muestreo teórico. Un guion semiestructurado orientó la recolección de datos. El análisis comparativo constante y concomitante siguió los pasos de codificación inicial y focalizada, con apoyo de memorandos, hasta la saturación teórica. El diagrama de Sankey integró el análisis de los datos, utilizando el *software* Atlas.ti.

37 participantes fueron ubicados en dos grupos muestrales. Se presenta la categoría “Experimentando la evolución tecnológica durante el proceso de envejecimiento” y dos subcategorías: “Experimentando sentimientos al envejecer frente al desarrollo tecnológico”; “Usando el *smartphone* y reconociendo nuevas experiencias”.

al experimentar el uso del *smartphone* , las personas mayores comprenden y reconocen la búsqueda de competencias digitales a partir de sus necesidades de utilidad y aplicabilidad de la tecnología de información y comunicación en su día a día, integrándola al proceso de envejecimiento saludable.

## Destacados

**(1)** Personas mayores aún viven segregadas de los jóvenes en relación al contexto tecnológico.

**(2)** Diariamente, personas mayores reconocen la necesidad, utilidad y aplicabilidad del *smartphone* .

**(3)** Las personas mayores están avanzando gradualmente en busca de competencias digitales.

**(4)** Las herramientas digitales son medios que potencian el envejecimiento saludable.

**(5)** Grupos de *WhatsApp* ^®^ ayudan a las personas mayores a comprometerse virtualmente con sus pares.

## Introducción

 El mundo ha experimentado dos transiciones importantes en los últimos años: el rápido envejecimiento de la población ^(^ 1 ^)^ , que invierte la pirámide demográfica y proyecta un aumento de personas mayores en relación con los jóvenes para las próximas décadas ^(^ 2 ^-^ 4 ^)^ , y la transición tecnológica, que abarca la universalización de las Tecnologías de Información y Comunicación (TIC) y gradualmente ha sustituido recursos y dispositivos analógicos por modelos digitales innovadores con acceso a Internet ^(^ 5 ^-^ 6 ^)^ . Con esto, la experiencia de las innovaciones tecnológicas genera cambios en las conexiones sociales con impactos en la salud, mediante la diseminación de información capaz de modificar comportamientos que afectan el aislamiento social, la soledad y la comunicación, especialmente en las personas mayores ^(^ 7 ^)^ . El uso del *smartphone* por las personas mayores en su día a día es impulsado por medio de significados que fortalecen los vínculos sociales, sentimientos y reconocimientos de las vivencias en pares en un contexto tecnológicamente conectado ^(^ 8 ^)^ . 

 Recientemente, hay interés científico sobre las potencialidades de las TIC como apoyo para el proceso inclusivo, saludable y activo de envejecimiento ^(^ 9 ^-^ 13 ^)^ y alineado a las propuestas de la Década del Envejecimiento Saludable (2021-2030) y a los Objetivos de Desarrollo Sostenible (ODS) ^(^ 2 ^-^ 4 ^,^ 11 ^)^ , afirmando que la tecnología digital es un agente transformador capaz de impulsar la independencia y productividad de las personas mayores ^(^ 1 ^)^ . 

 Como importante forma de comunicación, los *smartphones* , sus aplicaciones y las redes sociales están presentes en la vida de las personas ^(^ 13 ^)^ y en Brasil, en 2023, la posesión de este equipo en los domicilios (95%) superó, en todas las regiones, a la televisión tradicional (94%) y al teléfono fijo (12%) ^(^ 14 ^)^ . De poseer el dispositivo, el 75% de las personas mayores estadounidenses de 65 años acceden a la red ^(^ 15 ^)^ . 

 Es un hecho que existe una disparidad entre poseer el equipo y acceder funcionalmente a Internet. Diversos modelos conceptuales y teóricos han sido estudiados durante mucho tiempo ^(^ 16 ^-^ 17 ^)^ , perfeccionados y aplicados en relación con la aceptabilidad y aplicabilidad de la tecnología en el día a día de las personas mayores. Se busca contribuir con el despliegue que esto representa a medio y largo plazo en el envejecimiento ^(^ 1 ^,^ 17 ^-^ 18 ^)^ . Diferentes contextos son considerados para estrechar la discrepancia existente entre la aplicabilidad funcional de las TIC y simplemente poseer dispositivos como herramientas. Entre ellas, factores culturales, sentimientos, experiencias personales y motivaciones sociales se señalan como predictores de acceso ^(^ 19 ^)^ . 

 Las tecnologías digitales han sustituido y complementado a las analógicas reflejando cambios generacionales en los modelos de comunicación a lo largo de los años ^(^ 5 ^,^ 20 ^)^ . En los últimos años, las TIC han sido utilizadas como estrategias en los sistemas familiares para fomentar la comunicación, evitar rupturas sociales, repercutiendo positivamente y desarrollando nuevos roles en el cuidado entre sus miembros ^(^ 21 ^)^ . 

 Se destaca que el aislamiento social es una condición desfavorable que empobrece los vínculos y margina a las personas mayores, y que la mayoría de ellos disfrutan involucrarse en actividades sociales que aportan significado ^(^ 7 ^,^ 22 ^)^ . Al permitirse experimentar el mundo en red, las personas mayores pueden tener gestión de control de sus vidas por intermedio de Internet y sentirse pertenecientes a una comunidad ^(^ 23 ^)^ . 

 Estudios que analizaron propiedades de dispositivos y la utilización de las TIC por personas mayores verificaron que el acceso a Internet era más frecuente por personas mayores que utilizaban *smartphone* que aquellos que utilizaban *tablets* y computadoras ^(^ 11 ^,^ 24 ^)^ y que la conexión social puede ser promovida con esta herramienta, ofrecida de manera segura y viable ^(^ 22 ^)^ . 

 Al comprender la urgencia en la lucha contra el etarismo - cambio de la forma en que pensamos (estereotipos), cómo sentimos (prejuicios) y cómo actuamos (discriminación) ^(^ 4 ^)^ - surge la necesidad de dar visibilidad a la persona en envejecimiento comprometida digitalmente y que necesita apoyo, incluido el profesional, entendiendo sus sentimientos y anhelos. 

 Países como China ya estudian la necesidad de incentivar la actuación de la enfermería en la reducción de la exclusión digital, aumentando las competencias digitales entre las personas mayores ^(^ 25 ^)^ . El contexto brasileño aún carece de investigaciones que refuercen el alineamiento de la propuesta en utilizar las TIC para optimizar estrategias de educación en enfermería para la persona mayor. 

 Para ello, es extremadamente relevante conocer cómo es la adopción de los recursos tecnológicos por los protagonistas a medida que envejecen y los aplican a lo largo de sus vidas. Entonces, se cuestiona: ¿qué siente y experimenta la persona mayor que está envejeciendo al usar habitualmente recursos de un *smartphone* ? Y, se objetiva comprender los sentimientos y reconocimientos de personas mayores al experimentar el uso del *smartphone* en el cotidiano, así como las implicaciones en el proceso de envejecimiento saludable. 

## Método

### Diseño del estudio

 Investigación cualitativa, alineada al referencial conceptual del informe de línea base de la Década del Envejecimiento Saludable 2021-2030 ^(^ 2 ^,^ 4 ^)^ y utilizando como modelo teórico la *Unified Theory of Acceptance and Use of Technology* (UTAUT) ^(^ 16 ^)^ y el *Senior Technology Acceptance & Adoption Model* (STAM) ^(^ 17 ^)^ . La orientación metodológica que sustentó el estudio fue la Teoría Fundamentada en los Datos (TFD), en la vertiente Constructivista ^(^ 26 ^)^ . El rigor metodológico fue delineado a partir del *checklist Consolidated Criteria for Reporting Qualitative Research* (COREQ) ^(^ 27 ^)^ . 

### Escenario del estudio y selección de los participantes

 El escenario fue un proyecto de extensión comunitaria ^(^ 18 ^)^ , vinculado a una institución pública federal de enseñanza y que tiene una asociación con un centro municipal de convivencia para las personas mayores ubicado en la región sur de Brasil. El proyecto tiene como objetivo la promoción de competencias digitales y de salud, como el uso de *smartphones* por personas mayores, de manera orientada por profesionales, como recurso para proporcionar salud y bienestar e integrando las directrices que promueven el envejecimiento saludable ^(^ 19 ^)^ . 

 La selección de los potenciales participantes ocurrió intencionalmente basada en un conjunto de criterios de inclusión: a) personas de 60 años o más; b) usuarios de *smartphones* con acceso a Internet; c) participantes por al menos 6 meses del proyecto de extensión “Abuelos y Abuelas Conectados” ^(^ 18 ^)^ , d) y sin deterioro cognitivo. Para evaluar estos últimos criterios se utilizó el Mini Examen del Estado Mental (MEEM). Se incluyeron personas mayores con puntuación igual o superior a 27 puntos ^(^ 28 ^)^ . No hubo criterios de exclusión. 

 Se utilizó un enfoque de muestreo teórico, supuesto de la TFD ^(^ 26 ^,^ 29 ^)^ destinado a incluir un grupo diversificado de personas mayores con variedad de características y experiencias. Inicialmente, la investigadora principal estableció una relación con los potenciales participantes y en la secuencia dio andamiento a las demás etapas ( Figura 1 ). 

**Figura 1:** - Etapas del proceso de selección de los participantes (n = 37). Maringá, PR, Brasil, 2023

*Mini Examen del Estado Mental; ^†^ Consentimiento Informado

El reclutamiento de los participantes se realizó independientemente de sexo, raza o apoyo familiar, permitiendo una variación máxima de las características de los participantes. Como resultado, 37 personas participaron en el estudio y fueron organizadas en dos grupos muestrales, siendo el primer grupo muestral con 27 personas mayores con apoyo familiar y el segundo grupo incluyó a 10 personas mayores sin apoyo familiar, que vivían solos en sus casas.

 El alcance de la saturación teórica se significó a partir del momento en que ningún dato empírico adicional fue generado durante la fase de investigación conceptual, así fue determinado el muestreo teórico con el número de participantes ^(^ 26 ^)^ . El apoyo familiar mejora la calidad de vida relacionada con la salud física y mental de las personas mayores ^(^ 30 ^)^ y la satisfacción con la vida ^(^ 31 ^)^ . De este modo, relaciones significativas y positivas entre el apoyo social y el uso de *smartphones* ya evidenciadas en la literatura ^(^ 32 ^)^ proporcionaron la justificación para la creación de ambos grupos muestrales. Las directrices de tamaño de muestra recomiendan un rango de 20 a 30 participantes para alcanzar la saturación en un estudio fundamentado en los datos ^(^ 33 ^-^ 34 ^)^ . Aunque nuestra muestra estaba compuesta por 37 personas en total, en la 28ª entrevista la categoría central ya había sido identificada y dimensionada. 

### Procedimiento de recogida de datos

 La recogida de datos se llevó a cabo desde agosto de 2022 hasta marzo de 2023 utilizando llamadas comunes, por teléfono o teleconferencia ( *Google Meet* ), o vídeo mediante la aplicación *WhatsApp* ^®^ y solo estuvieron presentes en la llamada el investigador y el participante. 

El Consentimiento Informado (CI) y la autorización de imagen y voz, además de ser consentidas verbalmente, fueron firmadas y proporcionadas digitalmente al participante.

 Las entrevistas individuales fueron orientadas por una guía elaborada por los investigadores doctores del grupo de investigación NEAPE - Núcleo de Estudios Avanzados e Investigaciones sobre Envejecimiento - que aportaron su expertise en el área y, basándose en la literatura ^(^ 4 ^,^ 16 ^-^ 17 ^,^ 35 ^)^ . La guía se centró en las siguientes áreas: comprensión de la experiencia y vivencia de las personas mayores en el uso de Internet vía *smartphone* ; su utilidad percibida; facilitadores, sentimientos y constricciones en el uso de *smartphones* ; e implicaciones de las tecnologías en la salud y bienestar de las personas mayores. 

La aplicación de la guía en las dos primeras entrevistas semiestructuradas se evaluó en cuanto a la comprensibilidad, amplitud y lenguaje claro y conciso, y se realizaron adaptaciones de las preguntas conforme avanzaba la recogida de datos. Se integraron las dos entrevistas en el estudio y no fue necesario repetir ninguna de ellas.

La recogida de datos fue conducida por la investigadora principal, que recibió formación para la recogida y análisis simultáneo de datos, tiene experiencia en investigación cualitativa y con personas mayores usuarias de redes sociales virtuales y, con formación dual, en enfermería y gestión de TIC, favoreciendo una interacción diferenciada y acogedora en el momento de la entrevista.

 A partir de inferencias realizadas en el proceso de recogida de datos y en los análisis concomitantes, surgieron cuestiones relacionadas con el apoyo familiar en el domicilio de la persona mayor, que fueron tomadas notas y registradas en memorandos, según lo supuesto por la TFD ^(^ 26 ^)^ , y entonces se organizaron dos grupos muestrales distintos con estas características. 

 Las personas mayores que residen solas fueron incluidas en el estudio, ya que partieron de un contexto diferente al primer grupo, en el cual la persona mayor al compartir la cohabitación tiene más posibilidades de estar en un ambiente con recursos, herramientas y personas ambientadas con las tecnologías digitales, en especial el *smartphone* . 

 El cierre de la recogida se dio por saturación teórica, que es el punto en el cual no se genera ningún *insight* de nuevos conceptos y así se produjeron pruebas de forma repetida para conceptualizar la categoría principal ^(^ 29 ^)^ . 

 Se realizaron grabaciones de las entrevistas semiestructuradas con el consentimiento de los participantes, y estas fueron despersonalizadas en cuanto a su identificación, transcritas literalmente, utilizando el recurso *Reshape* ^®^ asistiendo en el mantenimiento de su integridad, y analizadas. El proceso de recogida y análisis de datos ocurrió simultáneamente. 

La duración de las entrevistas varió entre 31 y 72 minutos y tuvo, en promedio, una duración de 40 minutos. Reflexiones del investigador fueron registradas en formato de memorandos, que fueron redactados a lo largo de todo el proceso conforme progresaba la recogida, y fueron fundamentales para apoyar la integración, el relato de esquemas conceptuales, ilustrando el desarrollo de las ideas y códigos, dirigiendo la construcción de la teoría.

 No fue necesaria la devolución a los participantes para posibles correcciones y ajustes. Tras ser revisada toda la documentación con rigor, el material fue exportado al *software* Atlas.ti ^®^ . Las citas de los participantes fueron identificadas: numeradas secuencialmente; F o M (femenino o masculino), seguida por la caracterización de vivienda (solo o acompañado); edad en años. 

### Análisis de datos

 La codificación de los datos, según la TFD Constructivista ^(^ 26 ^)^ , se desarrolló en dos etapas, inicial y focalizada. En la primera, se exploró cada acción que proviene de los datos y se codificó incidente por incidente con el fin de comprender la información a partir de los significados y vivencias de los participantes, formando las primeras dimensiones del análisis de la experiencia. 

En la segunda etapa, focalizada, se realizó el análisis principal, más riguroso, que permite una comprensión analítica sobre el fenómeno promoviendo agrupamientos conceptuales hasta la identificación de categorías y subcategorías.

Ante todo el proceso, la retomada de los datos ocurre en el movimiento de comparación constante y, en este método, el investigador se desplaza entre el pensamiento inductivo y deductivo generando relación entre las categorías y articulación de la TFD entre interpretación e integración de nuevos datos.

El diagrama de Sankey, aquí utilizado representando la integración de los datos, es una figura clásica, que muestra la eficiencia energética del flujo de los datos y conexiones. Si los nudos tienen enlaces de entrada y de salida en el conjunto de nodos actualmente visible, serán colocados en franjas o capas intermedias, mostrando la fuerza de co-ocurrencia entre los pares de nudos. De forma simplificada, las franjas de cada lado del diagrama representan subcategorías conceptuales referentes a los datos analizados y nombrados y su anchura indica la eficiencia energética del flujo de datos (relaciones sustanciales cualitativas y cuantitativas entre las subcategorías cruzadas representadas por el testimonio de los participantes).

Cuanto mayor sea la anchura de la franja, más representación tiene el flujo de datos o intensidad de las relaciones sustanciales y, conforme hay cambio de color inicial de la franja, significa cruce con demás franjas, representando interacción entre flujos de datos de otras subcategorías, es decir, cruces entre temas.

### Rigor metodológico

 Los criterios de credibilidad, originalidad, resonancia y utilidad otorgaron rigor a la investigación y validez a la TFD ^(^ 26 ^)^ . La garantía de credibilidad se logró mediante un cuidado detallado con los datos durante la transcripción de las entrevistas; en la construcción de los memorandos que fueron comparados y contrastados constantemente, además del involucramiento prolongado del investigador para comprender profundamente el fenómeno que se estudia. El vínculo con cada participante promovió una relación armoniosa y de confianza, favoreciendo el intercambio de experiencias. Otra estrategia para aumentar la credibilidad fue alcanzar la saturación de los datos, cuando los participantes del estudio ya no proporcionaban información nueva ^(^ 36 ^)^ . 

 Para facilitar la discusión entre pares, la originalidad se evidenció mediante memorandos y representaciones esquemáticas del fenómeno asociadas al proceso reflexivo del investigador ^(^ 37 ^)^ . Se utilizó una combinación de análisis deductivo e inductivo, fomentada por el método TFD para asegurar la resonancia y crear conceptos que reflejan con precisión las experiencias de los participantes. 

Finalmente, la utilidad abarca la capacidad de profundizar en la comprensión de los participantes de la investigación sobre sus experiencias diarias, estableciendo una base para el desarrollo de políticas y buenas prácticas.

### Aspectos éticos

El proyecto fue aprobado por la Comisión Nacional de Ética en Investigación, bajo el dictamen nº 5.553.406 y CAAE: 57993522.0.0000.0104. Se observaron los preceptos éticos establecidos por las Resoluciones 466/2012 y 510/2016 del Consejo Nacional de Salud.

## Resultados

 Participaron en el estudio 37 personas mayores ( Figura 2 ) usuarios de *smartphone* por al menos 24 meses, que en su mayoría, acceden más de una hora diaria, denotando una considerable y significativa familiaridad con la tecnología que ofrece el dispositivo. 

 Para llegar al fenómeno, por medio de interacciones y relaciones entre las categorías y subcategorías, se permeó todo el conjunto de datos, unificando las diversas perspectivas y aspectos observados, proporcionando una comprensión amplia y completa. Los datos contemplan la categoría central y sus dos subcategorías ( Figura 3 ). 

**Figura 2:** - Caracterización de los participantes del estudio (n = 37). Maringá, PR, Brasil, 2023

*Desviación estándar; ^†^ Tiempo de pantalla autoreferido, referente al uso de *smartphone* en un período de 24 h

**Figura 3:** - Relación entre la Teoría Fundamentada en los Datos, Categorías y Subcategorías/Diagrama de Sankey. Maringá, PR, Brasil, 2023

*Fenómeno/ *Core Category* referente a la TFD (Tesis Doctoral); ^†^ Diagrama de Sankey ( Figura 4 )

 Las vivencias de las personas mayores al utilizar el *smartphone* , y cómo reconocer esas experiencias, se presentan en dos subcategorías: “Experimentando sentimientos al envejecer frente al desarrollo tecnológico” y “Usando el *smartphone* y reconociendo nuevas experiencias”. Para una visualización amplia y mejor comprensión de las co-ocurrencias, se agruparon/estandarizaron verbos representativos en cada subcategoría, que co-ocurrieron entre sí, siendo para la subcategoría 1 el verbo “sentir” y para la subcategoría 2, el verbo “reconocer”, representando las experiencias y el uso del *smartphone* , respectivamente, dentro del contexto y objetivo del estudio. 

 Ante los códigos analizados, con apoyo de recursos del *software* Atlas.ti ^®^ , fue posible identificar en cada categoría, las reacciones positivas/neutras y negativas en relación a la temática, y la codificación fue realizada por colores identificando cada una de esas reacciones. Las mayores interacciones identificadas a partir del cruce de las franjas explícitas en el diagrama de Sankey ( Figura 4 ) fueron evidenciadas en la interacción de reconocimiento de oportunidades para el envejecimiento y en la relación con las oportunidades de integración en el contexto tecnológico, con confianza en la búsqueda de información. 

**Figura 4:** - Integración de flujo de subcategorías “Experimentando sentimientos al envejecer frente al desarrollo tecnológico - Sentimientos” y “Usando el *smartphone* y reconociendo nuevas experiencias - Reconocimientos” de los participantes (n = 37). Maringá, PR, Brasil, 2023

*Diagrama de Sankey creado a partir del *software* Atlas.ti ^®^

 Ambas categorías, representadas en el diagrama, expresan las experiencias de los participantes, en cada una de las intersecciones y sus representaciones en el desarrollo a lo largo de sus vivencias, reflejan sobre el proceso de envejecimiento. Tras la inserción del *smartphone* en el cotidiano de estas personas mayores, modificaron algunas formas de pensar y actuar en relación a diversas situaciones, entre ellas, se destaca la fluidez con la que las informaciones llegan y los dejan actualizados en relación a diversos contextos, del local al global. 

 Participar de este proceso es una oportunidad que, para muchos, genera reconocimiento y sentimientos que los sitúan en el contexto social, individual e inculcando ideas que resuenan a largo plazo, inclusive la adopción de hábitos saludables. *[...] uno se queda más “al tanto” de las cosas que quiere saber y se entera en el momento, es muy bueno* (2-F-Sola, 67) *. [...] donde estoy, el celular está junto, si no tenía que esperar llegar a casa, llamar a alguien, o ir a la casa de las personas. Ahora no, con el celular en la mano me comunico, consulto cosas que necesito, hago todo* (26-F-Acompañada, 72) *. Uso el celular para recordar cosas básicas, por ejemplo, agua. Ahora mismo estoy con mi botella aquí al lado. Ya tuve infecciones crónicas de orina, no tenía el hábito de tomar agua* (30-F-Acompañada, 70). 

 Al conectarse en red, percibiendo estar más informados y aptos para buscar información, las personas mayores se reconocen más independientes y con mentalidad ajustada a los aspectos contemporáneos de la tecnología, aunque declaren necesidad de continuo aprendizaje. En sus sentimientos, explicitan la comprensión de lo que es real y de lo que es virtual. *[...] cuando trabajaba, no tenía Internet, pero tenía fax [...] vivimos de acuerdo con lo que está evolucionando en el mundo, si de aquí en adelante viene algo más allá de Internet, ella quedará en el pasado y me adaptaré a lo nuevo* (11-F-Sola, 68) *. [...] para mí no es difícil, no veo gran dificultad. Algunas que tengo, si voy tras ellas, aprendo* (28-F-Sola, 67). 

 En la perspectiva de participar, conciliar y alternar entre lo real y lo virtual, las personas mayores buscan apoyo en el modo virtual, por medio del *smartphone* , conectándose con personas, lugares, servicios e informaciones, reconocen este modo como una herramienta que puede ofrecer una gama de oportunidades, inclusive en el proceso de envejecimiento. 

 Las personas mayores, al comprender el ambiente virtual como una representación gráfica, algo transmutado y aplicado en situaciones reales, hacen proyecciones de futuro con sentimientos de optimismo a partir de influencias recibidas en red. *[...] cuando salgo, veo la dirección y ubicación en el mapa virtual. Para mí facilitó porque soy pésima para guardar nombres de calles, esta localización del celular me ayuda bastante* (10-F-Acompañada, 65) *. [...] Mira qué bien, estamos conversando y planeando (en línea) en grupo con cuatro amigas de infancia, una es de fuera de Brasil, vamos a hacer un ahorro para un viaje de crucero dentro de dos años. Entonces, esto es envejecer con sabiduría y salud. Es observar el futuro dentro de dos años, estaré viajando con mis amigas, es un optimismo que ya no tenía hace mucho tiempo* (20-F-Sola, 60) *.*

 Las personas entienden la posibilidad de envejecer conectadas en red como una oportunidad y privilegio en relación a los que no tienen el mismo acceso. *Ojalá todas las personas de nuestra edad tuvieran esta posibilidad, o interés en saber cosas de su salud, pues las posibilidades hoy en Internet no son tan difíciles como antes. Si tienen este interés... los ancianos pueden* (30-F-Acompañada, 70) *. [...] tengo la oportunidad de ejercitar mi mente, y envejecer mejor* (32-F-Acompañada, 60) *.*

 La fragmentación bajo la óptica de dos mundos, real y virtual, se complementan. Aunque la lucha contra el edadismo es un desafío de la década, las propias personas mayores cargan estigmas relacionados a la intitulación de sentirse pertenecientes a dos realidades, refiriéndose a la primera, una realidad más jovial y aceptada socialmente por la oportunidad de participar de este contexto tecnológico y la segunda, un segmento de personas jubiladas, pero insertas y comprometidas con las oportunidades ofrecidas por la tecnología. *[...] en cuanto a la tecnología solo digo que está siendo óptima para mí, envejecer en el mundo del modernismo es una gran oportunidad para nuestra generación* (22-F-Acompañada, 63) *. Usar Internet y el celular en este mundo lleno de tecnología, es importantísimo para mí, porque veo varias oportunidades de crecer por este medio* (37-M-Acompañado, 73) *. [...] no conseguía quedarme en casa cuando me jubilé, fue de repente, y el celular me ayudó mucho, me quedaba leyendo sobre varios temas y eso me ayudó a ser quien soy hoy, más jovial, pues después de los 60 es complicado, es difícil mirarse al espejo y ver que se llegó a una edad en la que las personas no te aceptan en todos los lugares con la misma atención de antes* (20-F-Sola, 60). 

 La sensación de bienestar se percibe ante la satisfacción de entender el envejecimiento como un proceso irreversible, pero susceptible de ser construido de forma saludable cuidando la salud de forma global. *Por más que participe en las actividades físicas en el centro de convivencia para prepararme para envejecer bien, llegará un momento en que estaré más limitado. Me siento bien usando el celular en este tiempo, sabiendo que tengo la capacidad de participar, aprender, tanto intelectual como físicamente* (22-F-Acompañada, 63) *. Estuve en una depresión profundísima casi llegando así a las vías de hecho, en este sentido el grupo me ayudó mucho con la parte social, empezar a conversar, escuchar, leer, videos de objetos antiguos que se pueden revisitar, parece que hace un rescate de nuestra alma, va allá al fondo del pozo y nos saca y dice… ¡mira todo esto que tienes de historia en tu vida, fue muy agradable y ha sido muy bueno! cambió mi vida* (20-F-Sola, 60) *.*

 Por otro lado, se sienten preocupados en relación a situaciones específicas, como estafas en el ambiente virtual que son comunes y victimizan a las personas mayores. El miedo a la exposición excesiva en redes sociales virtuales es un sentimiento que acompaña a las personas mayores conectadas en Internet en virtud de la divulgación de datos personales. *[...] la persona no sabe que ese mensaje es malicioso y lo pasa a la gente [...] también está la persona que tiene segundas intenciones, al pasar a un tercero, está cometiendo un delito* (8-M-Acompañado, 86) *. Soy cauteloso y desconfiado para cosas de dinero y banco, eso no hago nada* (12-M-Acompañado, 86) *. [...] no es falta de orientación (caer en la estafa), pero es un minuto de descuido y ya está* (22-F-Acompañada, 63) *. [...] tengo miedo porque allí (en red) casi no tenemos privacidad, no pongo fotos, no comento mis idas y venidas, me siento muy expuesto. En las redes sociales no participo, no me gusta toda esa exposición, prefiero más discreción* (10-F-Acompañada, 65) *.*

Al temer los peligros en Internet, reconocen y atribuyen fragilidades relacionadas a la limitación de los conocimientos tecnológicos, diferenciándose de los jóvenes que ya son nativos digitales y poseen aptitud en esta área inherente al desarrollo humano.

 Las personas mayores, incluso con limitaciones, reconocen que el nuevo modo de pensar, con el uso del *smartphone* , permite la participación y el compromiso en lo que ellos denominan “ritmo de la tecnología”. *[...] un niño toma el celular y lo manipula todo, nosotros tenemos más dificultad porque empezamos muy tarde* (1-F-Acompañada, 70) *. [...] la persona mayor tiene mucha importancia en la sociedad, a pesar de los pesares, vemos tanto prejuicio... pero todavía hay un objetivo, una mirada para este público anciano que está haciendo que la máquina gire también. Me siento “en el ritmo” formando parte del proceso tecnológico... Antes, a los 50 años, ya era una persona vieja y tonta, servía para cuidar nietos, hoy no, es muy diferente y lo encuentro loable, óptimo* (22-F-Acompañada, 63) *. Hoy, cuando abrí el documento que leímos (TCLE) hice clic en el lapicito, y apareció la firma que hice, [...] me sentí lista, recordé cómo se hacía y lo hice enseguida* (28-F-Sola, 67). *Siento que estoy acompañando el ritmo de la tecnología porque aprendo mucho e incluso enseño. Hay cosas que aprendí que mis nietos no sabían, como lo del micrófono negro, aprieta para hablar. Ellos no sabían eso, y yo se los enseñé* (29-F-Sola, 69) *.*

## Discusión

 Este estudio comprendió lo que la persona mayor siente y reconoce al utilizar el *smartphone* en su cotidiano y cómo esta experiencia influye en el proceso de envejecimiento saludable. Se diferencia de estudios anteriores que abordan a la persona mayor como aprendiz de tecnología e innova al centrarla como protagonista, usuaria de los recursos tecnológicos, y dotada de una nueva mentalidad en busca de competencias digitales para aplicarlas en su curso de vida. 

 Corroboramos estudios anteriores ^(^ 9 ^,^ 35 ^)^ , al señalar que las personas mayores, al percibir el *smartphone* como un beneficio en la vida diaria, lo han utilizado cada vez más en sus rutinas y esto se convierte en un factor determinante para su inclusión en las diversas actividades personales. Sin embargo, la disposición de las personas mayores para utilizar las tecnologías varía de acuerdo con su expectativa de provecho y la creencia de que estas les ayudan a mantenerse seguros ^(^ 16 ^-^ 17 ^,^ 35 ^,^ 38 ^)^ . 

 Traemos evidencias que demuestran que las personas mayores que se reconocen más independientes intelectualmente y autónomas en sus actividades diarias, al usar el *smartphone* . Se observa en otro estudio, que la mayoría de las teorías del envejecimiento no consideran la variabilidad individual, social o cultural de los objetivos y expectativas de las personas mayores ^(^ 38 ^)^ . 

 En el uso cotidiano o aplicabilidad de los avances tecnológicos en la vida de la persona mayor, la independencia es percibida como un factor importante para el envejecimiento exitoso ^(^ 38 ^)^ . Sin embargo, presentamos, en un contexto individualizado, los reconocimientos y sentimientos de la persona mayor comprometida tecnológicamente, y a partir de ello, corroborando nuestros resultados, entendemos que para la construcción de una vida significativa para las personas mayores es necesario pertenecer, reconocer, sentir. Así, nuestros resultados apuntaron a la interacción de varios sentimientos a partir de experiencias con el uso de la tecnología del *smartphone* , y la ocurrencia cruzada entre el reconocimiento de estos sentimientos y sus representaciones en el desarrollo a lo largo de sus experiencias, propician el envejecimiento saludable conforme propone el diagrama de Sankey. 

 En relación a la utilidad percibida desde el punto de aceptación de la tecnología propuesto por el modelo UTAUT ^(^ 16 ^)^ y la teoría STAM ^(^ 17 ^)^ , la persona mayor necesita considerar la aplicabilidad de la tecnología y sus herramientas en el cotidiano. Claramente, se observa en este estudio que, aunque utilice funcionalmente el *smartphone* y esto le satisfaga, es necesario estar en la dinámica del aprendizaje continuo. Las experiencias que fundamentan los sentimientos de las personas mayores al utilizar los *smartphones* están relacionadas a su propia percepción de competencia y confianza para manejar el equipo ^(^ 22 ^)^ . 

 En China, un estudio con personas mayores sobre los motivos de uso del *smartphone* señaló que la comunicación y la búsqueda de información influyen en la satisfacción con la vida y, esto hizo una mediación total con la alfabetización digital ^(^ 39 ^)^ , reafirmado por nuestros resultados. 

 Por consiguiente, es importante comprender que los factores sociales, como el apoyo familiar y de los pares y, los patrones culturales, ayudan en la adquisición de competencias digitales por las personas mayores, y su utilización en la vida cotidiana se refleja en cambios que promueven el envejecimiento saludable ^(^ 1 ^)^ . 

 Más allá del apoyo informal, profesionales de las más diversas áreas pueden ayudar a las personas mayores, especialmente los profesionales de la salud. Exacerbando las barreras de acceso a los cuidados presenciales, ellos pueden recomendar contenidos y aplicaciones que contengan información de salud ^(^ 25 ^)^ . En el Reino Unido más de la mitad de las personas mayores con 65 años o más están dispuestas a utilizar recursos digitales para la salud. Sin embargo, la voluntad de utilizar aplicaciones de salud recomendadas por profesionales disminuye a medida que la edad avanza ^(^ 12 ^)^ . Estos datos refuerzan la urgencia de tratar temas de esta temática con personas mayores de más edad, entendiendo sus necesidades. 

 En China se discute la prioridad que debe darse a remover algunos obstáculos en la oferta de herramientas, abarcando el diseño de los productos, que se concentra exclusivamente en los jóvenes y sus prioridades, el prejuicio relacionado a la edad y un ambiente externo que es anti-facilitador e inductivo. En otras palabras, las tecnologías son dirigidas conforme a la edad e infraestructura, pero deben ser repensadas de acuerdo a la necesidad del proceso de envejecimiento ^(^ 40 ^)^ . 

 Algunos descubrimientos indican que no hay unanimidad sobre los beneficios o perjuicios apropiados por las personas mayores mediante la adopción de la práctica recurrente del uso del *smartphone* y que hay una fusión para las influencias percibidas/reconocidas ^(^ 3 ^)^ . Este estudio mostró un sentimiento de optimismo, reflejado en sensaciones de bienestar percibido, que fue demostrado en el diagrama de Sankey en co-ocurrencia a otros sentimientos. Otras investigaciones reportan cuestiones positivas acerca de las influencias que las personas mayores reciben al utilizar el *smartphone* y aplicarlas en sus vidas ^(^ 3 ^,^ 41 ^-^ 42 ^)^ . 

 Los diversos sentimientos experimentados por las personas mayores en relación a la oportunidad de estar conectados en red con otras personas de la misma franja etaria y con el mundo digital generan experiencias de participación digital social, competencia técnica, mejora de la función física, cognitiva ^(^ 22 ^)^ y conexión con familiares inclusive en situaciones de vulnerabilidad ^(^ 21 ^)^ . Así también, trajimos evidencias de que las personas mayores se sienten privilegiadas frente a los pares que aún no han avanzado en el desarrollo de habilidades con herramientas digitales, confirmando resultados de otro estudio que apoya que el entrenamiento en tecnología tiene un impacto positivo en el uso de *smartphones* cuando se refiere al bienestar ^(^ 35 ^)^ . 

 El hecho de sentirse privilegiado no elimina la necesidad de examinar críticamente el modo de acceso de los demás que están bajo un prejuicio de marginación. Se destacan los resultados de un reciente estudio de revisión que aborda el prejuicio relacionado a la edad en los sistemas de inteligencia artificial, recorriendo los caminos del desarrollo tecnológico y reiterando la existencia de una segregación ^(^ 43 ^)^ . 

 El uso del *smartphone* promueve un acercamiento que atraviesa cuestiones etarias, proporcionando a las personas mayores la experiencia de pertenencia al mundo digital al participar activamente de las TIC ^(^ 38 ^)^ . 

Este estudio se alinea al marco conceptual de la Década del Envejecimiento Saludable, que trae como objeto de percepción y acción, la lucha contra el edadismo. Según la Organización Panamericana de la Salud, el edadismo es una discriminación por edad que resulta en una salud más precaria, aislamiento social, muertes prematuras además de generar un gasto de billones a las arcas públicas a nivel global.

 Evidenciamos pensamientos de las propias personas mayores participantes, que reconocen algunos factores que condicionan o restringen la competencia digital en relación a los jóvenes que son nativos de este tiempo. En todo el mundo, a ejemplo de países asiáticos como Japón, Singapur y Tailandia ^(^ 1 ^)^ , América del Norte ^(^ 15 ^)^ , de América del Sur, como Brasil ^(^ 14 ^)^ existen divergencias en relación a la adopción de tecnología por medio del *smartphone* entre jóvenes y personas mayores, aunque se haya percibido una disminución de la brecha existente entre los extremos etarios con el pasar de las décadas. 

 Nuestro estudio señala que las personas mayores poseen sentimientos de integración y pertenencia alineados a los conceptos innovadores a medida que surgen y se desarrollan. Así, estigmas y vergüenzas traídos por las propias personas contribuyen al envejecimiento activo y exitoso, incluyendo el prejuicio de edad en relación al uso y diseño de la tecnología digital ^(^ 38 ^)^ . En este sentido, productos desarrollados especialmente “para” y “por” personas mayores promueven la disminución del prejuicio de edad. 

 Miedos relacionados a la inseguridad, incertidumbre y recelos son comunes entre personas mayores que utilizan herramientas digitales para la comunicación. Como contribución negativa, destacamos que las estafas virtuales y el miedo a la exposición excesiva pueden llevar a las personas mayores a retraerse en las redes sociales virtuales, convirtiéndose en un factor impeditivo para una participación activa ^(^ 42 ^,^ 44 ^)^ . 

 Existe un sentimiento de miedo implicado en el reconocimiento de la presencia en un nuevo contexto de comunicación, con limitaciones naturales del proceso de transición. Corroborando esto, algunas barreras dificultan el uso de la tecnología del *smartphone* por personas mayores, incluyendo miedo, falta de conocimiento, preocupación en relación a la complejidad, habilidad física, y dificultad de comunicación ^(^ 35 ^)^ . Se resalta que las aplicaciones virtuales no deben ser desarrolladas específicamente para públicos específicos, como las personas mayores, pues esto resulta en facilidad de adaptación y aprendizaje, lo que, consecuentemente, implica en la permanencia de uso de las herramientas y dispositivos virtuales a mediano y largo plazo ^(^ 9 ^,^ 45 ^)^ . 

 Los hallazgos apuntan a fragilidades que se interceptan con la restricción de conocimientos tecnológicos. Incluso con estas restricciones, se percibieron sentimientos de compromiso tecnológico por parte de las personas mayores, fortaleciendo resultados de otros estudios ^(^ 35 ^,^ 38 ^)^ , que señalan que algunos factores son facilitadores y otros dificultadores en el uso de formas que diversifiquen el apoyo institucional y familiar para la orientación del desarrollo personal con nuevas tecnologías, además de las motivaciones y experiencias frente al proceso de envejecimiento ^(^ 38 ^)^ . 

 Estudiamos la percepción y sentimientos de las personas mayores saludables, sin embargo, la literatura trae datos sobre asociaciones del uso desmedido y excesivo de la tecnología, especialmente del *smartphone* por personas mayores, que impactan en la calidad del sueño debido a factores de estrés y ansiedad ^(^ 46 ^)^ . 

 De sobremanera e intencionalmente, el desarrollo de las competencias digitales y la formación tecnológica para el público de personas mayores necesitan originarse en las intenciones y comportamientos de uso. Esto posibilitará el desarrollo de modelos de aceptación en estudios futuros ^(^ 35 ^)^ . 

 Identificamos limitaciones en este estudio relacionadas a la dirección de evidencias solo para personas mayores que ya poseen contacto con TIC, incorporando *smartphones* en sus rutinas y aún, orientadas por profesionales para tal uso, en un proyecto de extensión. No se consideró la intencionalidad de aquellas personas mayores que aún desean integrarse, pero que no tuvieron oportunidad. Se sugiere explorar esta temática en nuevas investigaciones y perspectivas más diversificadas en términos étnicos, en un abordaje basado en la comunidad, por ejemplo, lo que podrá llevar a una mejor comprensión del impacto de las TIC en el envejecimiento saludable. 

Tanto bajo la óptica de la inclusión social digital, como bajo la óptica de la promoción de la salud este artículo innova al traer la percepción de los efectos que el uso de la tecnología incide en la vida de la persona mayor partiendo de las experiencias de su curso de vida y motivaciones personales, proporciona subsidios para el desarrollo de estrategias que pueden ser guiadas por profesionales de la salud y propuestas para la comunidad y para el público de personas mayores. La alineación de la búsqueda por competencias digitales al cotidiano actúa en el combate al edadismo y en el envejecimiento saludable, a largo plazo contribuyendo con ahínco al avance del conocimiento científico para el área de la salud y enfermería.

## Conclusión

 La comprensión de los sentimientos y reconocimientos de las personas mayores en relación al uso de la tecnología del *smartphone* en el cotidiano nos hace vislumbrar que, a veces, las personas mayores, aún viven segregadas de los jóvenes en el contexto de la tecnología. Incluso sintiéndose integradas, reconociendo una nueva mentalidad y oportunidad de optimización de la independencia al utilizar el *smartphone* , las experiencias de las personas mayores revelan una fragmentación entre lo real y lo virtual. Sin embargo, este hecho no es determinante para que este proceso sea estático. Gradualmente, mediante el reconocimiento de sus necesidades en relación a la tecnología, principalmente de TIC, la percepción de la utilidad y aplicabilidad en el cotidiano, la identificación con pares, la búsqueda por competencias digitales ha convertido a la persona mayor en protagonista del aprendizaje, reverberando en el transcurso de su envejecimiento. 

 La permanencia de las personas mayores en el compromiso virtual destaca condiciones favorables para la promoción de la salud en el proceso de envejecimiento. La posesión del *smartphone* no garantiza su uso, ni la inclusión social digital, pero los estímulos profesionales en grupos virtuales y las orientaciones sobre prácticas saludables los integran en un ambiente de recursos e informaciones aplicables al proceso de envejecimiento saludable. 

Adicionalmente, la enfermería tiene condiciones de contribuir en el proceso de toma de decisiones en el campo virtual, pudiendo sobresalir como liderazgo, conduciendo estrategias que cambien la realidad conceptual y práctica del edadismo.

# Translation: Uso de *smartphone* por pessoas idosas no processo de envelhecimento saudável: uma teoria fundamentada nos dados *

**DOI:** 10.1590/1518-8345.7252.4384

**Published:** 2024-10-10

**Authors:** Adriana Martins Gallo, Juliane Pagliari Araujo, Wanessa Cristina Baccon, Francielle Renata Danielli Martins Marques, Maria Aparecida Salci, Lígia Carreira

**Affiliations:** ^1^ Universidade Estadual de Maringá, Maringá, PR, Brasil; ^2^ Instituto Federal do Paraná, Astorga, PR, Brasil; ^3^ Bolsista da Coordenação de Aperfeiçoamento de Pessoal de Nível Superior (CAPES), Brasil; ^4^ Universidade Estadual de Londrina, Londrina, PR, Brasil; ^5^ Instituto Federal do Paraná. Londrina, PR, Brasil

**Keywords:** Idoso, Envelhecimento Saudável, Teoria Fundamentada nos Dados, Smartphone, Promoção de Saúde, Mídias Sociais

## Abstract

compreender os sentimentos e reconhecimentos de pessoas idosas ao experienciarem o uso do *smartphone* no cotidiano, bem como as implicações no processo de envelhecimento saudável.

pesquisa qualitativa, utilizando *Unified Theory of Acceptance and Use of Technology* ; *Senior Technology Acceptance & Adoption Model* e o referencial metodológico Teoria Fundamentada nos Dados, na vertente Construtivista. Recrutou-se intencionalmente pessoas com 60 anos ou mais; usuários de *smartphones* ; participantes há seis meses ou mais de um projeto de extensão, sem comprometimento cognitivo, determinados por amostragem teórica. Um roteiro semiestruturado orientou a coleta de dados. A análise comparativa constante e concomitante seguiu os passos de codificação inicial e focalizada, com apoio de memorandos, até a saturação teórica. O diagrama de Sankey integrou a análise dos dados, utilizando o *software* Atlas.ti.

37 participantes foram alocados em dois grupos amostrais. Apresenta-se a categoria “Experimentando a evolução tecnológica durante o processo de envelhecimento” e duas subcategorias: “Experimentando sentimentos ao envelhecer frente ao desenvolvimento tecnológico”; “Usando o *smartphone* e reconhecendo novas experiências”.

ao experienciar o uso do *smartphone* , pessoas idosas compreendem e reconhecem a busca por competências digitais partindo de suas necessidades de utilidade e aplicabilidade das Tecnologias de Informação e Comunicação em seu cotidiano, integrando ao processo de envelhecimento saudável.

## Destaques

**(1)** Pessoas idosas ainda vivem segregadas dos jovens em relação ao contexto tecnológico.

**(2)** Cotidianamente, pessoas idosas reconhecem necessidade, utilidade e aplicabilidade do *smartphone* .

**(3)** As pessoas idosas estão se movendo gradualmente em busca de competências digitais.

**(4)** Ferramentas digitais são meios potencializadores do envelhecimento saudável.

**(5)** Grupos de *WhatsApp* ^®^ auxiliam os idosos a se engajarem virtualmente com pares.

## Introdução

 O mundo tem vivenciado duas transições importantes nos últimos anos: o rápido envelhecimento da população ^(^ 1 ^)^ – invertendo a pirâmide demográfica e projetando um aumento de pessoas idosas em relação aos jovens para as próximas décadas ^(^ 2 ^-^ 4 ^)^ ; e a transição tecnológica – que engloba a universalização das Tecnologias de Informação e Comunicação (TIC), o que gradualmente tem substituído recursos e dispositivos analógicos por modelos digitais com acesso à Internet ^(^ 5 ^-^ 6 ^)^ . Com isso, a experiência das inovações tecnológicas gera alterações nas conexões sociais com impactos na saúde, por meio da disseminação de informações capazes de modificar comportamentos que afetam o isolamento social, a solidão e a comunicação, principalmente nas pessoas mais velhas ^(^ 7 ^)^ . O uso do *smartphone* pelas pessoas idosas em seu cotidiano é propelido por meio de significados fortalecedores de vínculos sociais, sentimentos e reconhecimentos das vivências em pares, num contexto tecnologicamente conectado ^(^ 8 ^)^ . 

 Recentemente, há interesse científico sobre as potencialidades das TICs como apoio para o processo inclusivo, sadio e ativo de envelhecimento ^(^ 9 ^-^ 13 ^)^ , alinhado às propostas da Década do Envelhecimento Saudável (2021-2030) e aos Objetivos do Desenvolvimento Sustentável (ODS) ^(^ 2 ^-^ 4 ^,^ 11 ^)^ , afirmando a tecnologia digital como um agente transformador, capaz de impulsionar a independência e a produtividade de pessoas idosas ^(^ 1 ^)^ . 

 Como importante forma de comunicação, os *smartphones* , seus aplicativos e as redes sociais se fazem presentes na vida das pessoas ^(^ 13 ^)^ . No Brasil, em 2023, a posse desse equipamento nos domicílios ultrapassou (com um percentual de 95%), em todas as regiões, a tradicional televisão (94%) e o telefone fixo (12%) ^(^ 14 ^)^ . De posse do dispositivo, 75% das pessoas idosas norte-americanas com 65 anos acessam a rede ^(^ 15 ^)^ . 

 É fato que há disparidade entre possuir o equipamento e acessar funcionalmente a Internet. Diversos modelos conceituais e teóricos têm sido estudados de longa data ^(^ 16 ^-^ 17 ^)^ , aperfeiçoados e aplicados em relação à aceitabilidade e aplicabilidade da tecnologia no cotidiano de pessoas idosas. Busca-se contribuir com o desdobramento que isso representa em médio e longo prazo no envelhecimento ^(^ 1 ^,^ 17 ^-^ 18 ^)^ . Diferentes contextos são considerados para o estreitamento da discrepância existente entre a aplicabilidade funcional das TICs e apenas portar dispositivos como ferramentas. Dentre elas, fatores culturais, sentimentos, experiências pessoais e motivações sociais são apontadas como preditores de acesso ^(^ 19 ^)^ . 

 As tecnologias digitais têm substituído e complementado as analógicas refletindo mudanças geracionais nos modelos de comunicação ao longo dos anos ^(^ 5 ^,^ 20 ^)^ . Nos últimos anos, as TICs foram utilizadas como estratégias nos sistemas familiares para promover comunicação, evitar rompimentos sociais, repercutindo positivamente e desenvolvendo novos papéis no cuidado entre seus membros ^(^ 21 ^)^ . 

 Ressalta-se que o isolamento social é condição desfavorável que empobrece os vínculos e marginaliza as pessoas idosas, já que a maioria delas gosta de se envolver em atividades sociais que tragam significados ^(^ 7 ^,^ 22 ^)^ . Ao se permitirem experienciar o mundo em rede, as pessoas idosas podem ter gestão de controle de suas vidas por meio da Internet e sentirem-se pertencentes a uma comunidade ^(^ 23 ^)^ . 

 Estudos que analisaram propriedades de dispositivos e a utilização das TICs por pessoas idosas verificaram que o acesso à Internet era mais frequente entre pessoas idosas que utilizavam *smartphone* do que entre as que utilizavam *tablets* e computadores ^(^ 11 ^,^ 24 ^)^ e que a ligação social pode ser promovida com essa ferramenta, oferecida de modo seguro e viável ^(^ 22 ^)^ . 

 Ao compreender a urgência na luta contra o etarismo – mudança da forma como pensamos (a partir de estereótipos), como sentimos (preconceitos) e como agimos (pela discriminação) ^(^ 4 ^)^ – surge a necessidade de dar visibilidade à pessoa em envelhecimento engajada digitalmente e que necessita de apoio, inclusive profissional, entendendo seus sentimentos e anseios. 

 Países como a China já estudam a necessidade de incentivar a atuação da enfermagem na redução da exclusão digital, aumentando as competências digitais entre as pessoas idosas ^(^ 25 ^)^ . O contexto brasileiro ainda carece de pesquisas que reforcem o alinhamento da proposta em utilizar as TICs para otimizar estratégias de educação em enfermagem para a pessoa idosa. 

 Para isso, é extremamente relevante conhecer como é a adoção dos recursos tecnológicos pelos protagonistas à medida que envelhecem e a forma como são aplicados ao longo de suas vidas. Então, questiona-se: o que sente e experimenta a pessoa idosa que está a envelhecer ao usar habitualmente os recursos de um *smartphone* ? Objetiva-se, assim, compreender os sentimentos e reconhecimentos de pessoas idosas ao experienciarem o uso do *smartphone* no cotidiano, bem como as implicações no processo de envelhecimento saudável. 

## Método

### Delineamento do estudo

 Trata-se de uma pesquisa qualitativa alinhada ao referencial conceitual do relatório de linha de base da Década do Envelhecimento Saudável 2021-2030 ^(^ 2 ^,^ 4 ^)^ e que utiliza como modelo teórico a *Unified Theory of Acceptance and Use of Technology* (UTAUT) ^(^ 16 ^)^ e o *Senior Technology Acceptance & Adoption Model* (STAM) ^(^ 17 ^)^ . A orientação metodológica que sustentou o estudo foi a Teoria Fundamentada nos Dados (TFD), na vertente Construtivista ^(^ 26 ^)^ . O rigor metodológico foi delineado a partir do *checklist* *Consolidated Criteria- for Reporting Qualitative Research* (COREQ) ^(^ 27 ^)^ . 

### Cenário do estudo e seleção dos participantes

 O cenário foi um projeto de extensão comunitária ^(^ 18 ^)^ , vinculado a uma instituição pública federal de ensino e que tem parceria com um centro municipal de convivência para as pessoas idosas localizado na região Sul do Brasil. O projeto tem como objetivo a promoção de competências digitais e de saúde, a exemplo do uso de *smartphones* por pessoas idosas, de forma orientada por profissionais, como recurso para proporcionar saúde e bem-estar, integrando as diretrizes que promovem o envelhecimento saudável ^(^ 19 ^)^ . 

 A seleção dos potenciais participantes ocorreu intencionalmente baseada em um conjunto de critérios de inclusão: a) pessoas com 60 anos ou mais; b) usuários de *smartphones* com acesso à Internet; c) participantes por pelo menos 6 meses do projeto de extensão “Vovôs e Vovós Conectados” ^(^ 18 ^)^ e d) sem comprometimento cognitivo. Para avaliar o último critério foi utilizado o Mini Exame do Estado Mental (MEEM). Foram incluídas pessoas idosas com pontuação igual ou superior a 27 pontos ^(^ 28 ^)^ . Não houve critérios de exclusão. 

 Foi utilizada uma abordagem de amostragem teórica, pressuposto da TFD ^(^ 26 ^,^ 29 ^)^ destinada a incluir um grupo diversificado de pessoas idosas com variedade de características e experiências. Inicialmente, a pesquisadora principal estabeleceu um relacionamento com os potenciais participantes e na sequência deu andamento às demais etapas (Figura 1). 

**Figura 1:** - Etapas do processo de seleção dos participantes (n = 37). Maringá, PR, Brasil, 2023

*Mini Exame do Estado Mental; ^†^ Termo de Consentimento Livre e Esclarecido

O recrutamento dos participantes ocorreu independentemente de sexo, raça ou apoio familiar, permitindo variação máxima das características dos participantes. Como resultado, 37 pessoas participaram do estudo e foram organizadas em dois grupos amostrais, sendo o primeiro composto por 27 pessoas idosas com apoio familiar e o segundo grupo incluiu 10 pessoas idosas sem apoio familiar, que viviam sozinhos em suas casas.

 O alcance da saturação teórica foi significado a partir do momento que nenhum dado empírico adicional foi gerado durante a fase de investigação conceitual, assim foi determinada a amostragem teórica com o número de participantes ^(^ 26 ^)^ . O apoio familiar melhora a qualidade de vida relacionada à saúde física e mental de pessoas idosas ^(^ 30 ^)^ e a satisfação com a vida ^(^ 31 ^)^ . Desse modo, as relações significativas e positivas entre apoio social e uso de *smartphones* , já evidenciadas na literatura ^(^ 32 ^)^ , forneceram a justificativa para a criação de ambos os grupos amostrais. As diretrizes de tamanho de amostra recomendam uma faixa de 20 a 30 participantes para atingir a saturação em um estudo fundamentado nos dados ^(^ 33 ^-^ 34 ^)^ . Embora a nossa amostra fosse composta por 37 pessoas no total, na 28ª entrevista a categoria central já tinha sido identificada e dimensionalizada. 

### Procedimento de coleta de dados

 A coleta de dados ocorreu no período de agosto de 2022 a março de 2023, utilizando ligação comum, via telefone ou teleconferência ( *meet* ) ou, ainda, ligação de vídeo por meio do aplicativo *WhatsApp* ^®^ . Na chamada, estavam presentes apenas a pesquisadora e o participante. 

O Termo de Consentimento Livre e Esclarecido (TCLE) e a autorização de imagem e voz, além de consentidos verbalmente, foram assinados e disponibilizados digitalmente ao participante.

 As entrevistas individuais foram orientadas por um guia elaborado pelos pesquisadores doutores do grupo de pesquisa NEAPE – Núcleo de Estudos Avançados e Pesquisas sobre Envelhecimento –, que contribuíram com sua expertise na área e, com base na literatura ^(^ 4 ^,^ 16 ^-^ 17 ^,^ 35 ^)^ . O guia foi focado nas seguintes áreas: compreensão da experiência e vivência de pessoas idosas no uso da Internet via *smartphone* ; sua utilidade percebida; facilitadores, sentimentos e constrangimentos na utilização de *smartphones* ; implicações das tecnologias na saúde e no bem-estar de pessoas idosas. 

A aplicação do guia, nas duas primeiras entrevistas semiestruturadas, foi avaliada quanto à compreensibilidade, abrangência e linguagem clara e concisa, bem como considerando as adaptações das questões realizadas conforme o andamento da coleta. Houve a integração das duas entrevistas no estudo e não foi necessário repetir nenhuma delas.

A coleta de dados foi conduzida pela pesquisadora principal, que recebeu treinamento para coleta e análise simultânea de dados, tem experiência em pesquisa qualitativa e com pessoas idosas usuárias de redes sociais virtuais, além de dupla formação, em enfermagem e gestão de TICs, favorecendo uma interação diferenciada e acolhedora no momento da entrevista.

 A partir de inferências realizadas no processo de coleta de dados e nas análises concomitantes, emergiram questões relacionadas ao apoio familiar no domicílio da pessoa idosa, que foram tomadas como notas e registradas em memorandos, conforme pressupostos pela TFD ^(^ 26 ^)^ . Assim, organizou-se dois grupos amostrais distintos com tais características. 

 Pessoas idosas que residem desacompanhadas foram incluídas no estudo, visto que se partiu de um contexto diferente do primeiro grupo, no qual a pessoa idosa, ao compartilhar a coabitação, tem mais chances de estar em um ambiente com recursos, ferramentas e pessoas ambientadas com as tecnologias digitais, em especial o *smartphone.*

 O encerramento da coleta se deu por saturação teórica, isto é, trata-se do ponto no qual nenhum *insight* de novos conceitos é gerado, sendo assim, foram produzidas provas de forma repetida para conceituar a principal categoria ^(^ 29 ^)^ . 

 Realizadas as gravações das entrevistas semiestruturadas com o consentimento dos participantes, procedeu-se à descaracterização quanto à identificação das entrevistas, as quais foram transcritas literalmente, utilizado o recurso *Reshape* ^®^ , auxiliando na manutenção de sua integralidade, de forma que pudessem ser analisadas. O processo de coleta e análise de dados ocorreu simultaneamente. 

A duração das entrevistas variou entre 31 e 72 minutos e teve, em média, a duração de 40 minutos. As reflexões da pesquisadora foram registradas em formato de memorandos, que foram redigidos ao longo de todo o processo conforme progredia a coleta, sendo fundamentais para apoiar a integração, o relato de esquemas conceituais, ilustrando o desenvolvimento das ideias e códigos, direcionando a construção da teoria.

 Não houve necessidade de devolução aos participantes para possíveis correções e ajustes. Após a documentação ser conferida com todo o rigor, o material foi exportado para o *software* Atlas.ti ^®^ . As citações dos participantes foram identificadas: numeradas sequencialmente; designadas por F ou M (a depender se o gênero do entrevistado era feminino ou masculino), sucedida pela caracterização de moradia (sozinho ou acompanhado); além da idade em anos. 

### Análise de dados

 A codificação dos dados, segundo a TFD Construtivista ^(^ 26 ^)^ , foi desenvolvida em duas etapas: a inicial e a focalizada. Na primeira, explorou-se cada ação que provém dos dados e codificou-se incidente por incidente a fim da compreensão das informações a partir dos significados e vivências dos participantes, formando as primeiras dimensões da análise da experiência. 

Na segunda etapa, isto é, a focalizada, deu-se a análise principal, mais rigorosa, possibilitando uma compreensão analítica sobre o fenômeno promovendo agrupamentos conceituais até a identificação de categorias e subcategorias.

Diante de todo o processo, a retomada dos dados ocorre no movimento de comparação constante, de forma que o pesquisador se desloca entre o pensamento indutivo e dedutivo, gerando relação entre as categorias e articulação da TFD entre interpretação e integração de novos dados.

 O diagrama de Sankey, aqui utilizado para representar a integração dos dados, é uma figura clássica, que mostra a eficiência energética do fluxo dos dados e conexões. Se os nós tiverem *links* de entrada e de saída no conjunto de nós atualmente visível, eles serão colocados em faixas ou camadas intermediárias, mostrando a força de co-ocorrência entre os pares de nós. De forma simplificada, as faixas de cada lado referentes aos do diagrama representam subcategorias conceituais dados analisados e nomeados, sua largura indica, assim, a eficiência energética do fluxo de dados (relações substanciais qualitativas e quantitativas entre as subcategorias cruzadas representadas pelo depoimento dos participantes). 

Quanto maior a largura da faixa, mais representação tem o fluxo de dados ou intensidade das relações substanciais, além disso, a mudança da cor inicial da faixa significa cruzamento com demais faixas, representando interação entre fluxos de dados de outras subcategorias, ou seja, os cruzamentos entre temas.

### Rigor metodológico

 Os critérios de credibilidade, originalidade, ressonância e utilidade conferiram o rigor da pesquisa e a validade da TFD ^(^ 26 ^)^ . A garantia da credibilidade se deu pelo cuidado detalhado com os dados durante a transcrição das entrevistas; na construção dos memorandos comparados e contrastados constantemente, além do envolvimento prolongado da pesquisadora para compreender profundamente o fenômeno que está sendo estudado. O vínculo com cada participante promoveu uma relação harmoniosa e de confiança favorecendo o compartilhamento de experiências. Outra estratégia para aumentar a credibilidade foi atingir a saturação dos dados, quando os participantes do estudo não mais forneciam informações novas ^(^ 36 ^)^ . 

 Para facilitar a discussão entre os pares, a originalidade foi evidenciada por meio de memorandos e representações esquemáticas do fenômeno associadas ao processo reflexivo do pesquisador ^(^ 37 ^)^ . Utilizou-se, como já mencionado, uma combinação de análise dedutiva e indutiva, fomentada pelo método TFD para assegurar a ressonância e criar conceitos que refletem com precisão as experiências dos participantes. 

Finalmente, a utilidade abrange a capacidade de aprofundar a compreensão dos participantes da investigação sobre as suas experiências diárias, o estabelecimento de uma base para o desenvolvimento de políticas e boas práticas.

### Aspectos éticos

O projeto foi aprovado pela Comissão Nacional de Ética em Pesquisa, sob parecer nº 5.553.406 e CAAE: 57993522.0.0000.0104. Observou-se os preceitos éticos estabelecidos pelas Resoluções 466/2012 e 510/2016 do Conselho Nacional de Saúde.

## Resultados

 Participaram do estudo 37 pessoas idosas (Figura 2) usuárias de *smartphone* há pelo menos 24 meses, as quais, em maioria, acessam mais de uma hora diária, denotando uma familiaridade considerável expressiva com a tecnologia que o dispositivo oferece. 

Para se chegar ao fenômeno, por meio de interações e relações entre as categorias e subcategorias, permeou-se todo o conjunto de dados, unificando as diversas perspectivas e aspectos observados, proporcionando uma compreensão abrangente e completa. Os dados contemplam a categoria central e suas duas subcategorias (Figura 3).

**Figura 2:** - Caracterização dos participantes do estudo (n = 37). Maringá, PR, Brasil, 2023

*Desvio-padrão; ^†^ Tempo de tela autorreferido, referente ao uso de *smartphone* em um período de 24 h

**Figura 3:** - Relação entre a Teoria Fundamentada nos Dados, Categorias e Subcategorias/Diagrama de Sankey. Maringá, PR, Brasil, 2023

*Fenômeno/ *Core Category* referente a TFD (Tese de Doutorado); ^†^ Diagrama de Sankey (Figura 4)

 As vivências das pessoas idosas ao utilizarem o *smartphone* e a forma como reconhecem essas experiências estão apresentadas em duas subcategorias: “Experimentando sentimentos ao envelhecer frente ao desenvolvimento tecnológico” e “Usando o *smartphone* e reconhecendo novas experiências”. Para uma visualização ampla e melhor compreensão das co-ocorrências, foram agrupados/padronizados os verbos representativos em cada subcategoria, que co-ocorreram entre si, sendo que a subcategoria 1 foi agrupada pelo uso do verbo “sentir” e, para a subcategoria 2, considerou-se o verbo “reconhecer”, representando as experiências e o uso do *smartphone* , respectivamente, dentro do contexto e objetivo do estudo. 

 Diante dos códigos analisados, com apoio de recursos do *software* Atlas.ti ^®^ , foi possível identificar, em cada categoria, as reações positivas/neutras e negativas em relação à temática, assim como a codificação realizada por cores identificando cada uma dessas reações. As maiores interações identificadas a partir do cruzamento das faixas explícitas no diagrama de Sankey (Figura 4) foram evidenciadas na interação de reconhecimento de oportunidades para o envelhecimento e na relação com as oportunidades de integração no contexto tecnológico, com confiança na busca de informação. 

**Figura 4:** - Integração de fluxo de subcategorias “Experimentando sentimentos ao envelhecer frente ao desenvolvimento tecnológico - Sentimentos” e “Usando o *smartphone* e reconhecendo novas experiências - Reconhecimentos” dos participantes (n = 37). Maringá, PR, Brasil, 2023

*Diagrama de Sankey criado a partir do *software* Atlas.ti ^®^

 Ambas as categorias representadas no diagrama expressam as experiências dos participantes, cada uma das intercepções e suas representações no desdobramento ao longo de suas vivências refletem o processo de envelhecimento. Após a inserção do *smartphone* no cotidiano dessas pessoas, algumas formas de pensar e agir se modificaram em relação a diversas situações, dentre elas, destaca-se a fluidez com que as informações chegam e os deixam atualizados em relação a diversos contextos, do local ao global. 

 Participar desse processo é uma oportunidade que, para muitos, gera reconhecimento e sentimentos que os situam no contexto social e individual, incutindo ideias que ressoam, a longo prazo, inclusive na adoção de hábitos saudáveis. *[...] a gente fica mais “a par” das coisas que quer saber e fica sabendo na hora, é muito bom* (2-F-Sozinha, 67). *[...] onde estou, o celular está junto, senão tinha que esperar chegar em casa, ligar para alguém, ou ir na casa das pessoas. Agora não, com o celular na mão me comunico, consulto coisas que preciso, faço tudo* (26-F-Acompanhada, 72). *Uso celular para lembrar coisas básicas, por exemplo, água. Agora mesmo estou com minha garrafa aqui do lado. Já tive infecções crônicas de urina, não tinha hábito de tomar água* (30-F-Acompanhada, 70). 

 Ao conectar-se em rede, percebendo estar mais informadas e aptas para buscar informações, as pessoas idosas se reconhecem mais independentes e com a mentalidade ajustada aos aspectos contemporâneos da tecnologia, mesmo que declarem necessidade de contínuo aprendizado. Em seus sentimentos, explicitam a compreensão do que é real e do que é virtual. *[...] quando trabalhava, não tinha Internet, mas tinha fax [...] vivemos de acordo com o que está evoluindo no mundo, se daqui para frente vir algo além da Internet, ela ficar no passado e vou me adaptar ao novo* (11-F-Sozinha, 68). *[...] para mim não é difícil, não vejo grande dificuldade. Algumas que eu tenho, se for atrás, eu aprendo* (28-F-Sozinha, 67). 

 Na perspectiva de participar, conciliar e alternar entre o real e virtual, as pessoas idosas buscam apoio no modo virtual, pelo *smartphone* , conectando-se com pessoas, lugares, serviços e informações, reconhecendo esse modo de interação como uma ferramenta que pode oferecer uma gama de oportunidades, inclusive no processo de envelhecimento. 

 As pessoas idosas, ao compreenderem o ambiente virtual como uma representação gráfica, algo transmutado e aplicado em situações reais, fazem projeções de futuro com sentimentos de otimismo a partir de influências recebidas em rede. *[...] quando saio, vejo o endereço e localização no mapa virtual. Para mim facilitou porque sou péssima em guardar nomes de ruas, essa localização do celular me ajuda bastante* (10-F-Acompanhada, 65). *[...] Olha que legal, estamos conversando e planejando (online) em grupo com quatro amigas de infância, uma é de fora do Brasil, vamos fazer uma poupança para uma viagem de cruzeiro daqui dois anos. Então, isso é envelhecer com sabedoria e saúde. É observar o futuro daqui dois anos, estarei viajando com as minhas amigas, é um otimismo que já não tinha há muito tempo* (20-F-Sozinha, 60). 

 Elas entendem a possibilidade de envelhecer conectadas em rede como uma oportunidade e privilégio em relação aos que não têm o mesmo acesso. *Quem dera se todas pessoas da nossa idade tivessem essa possibilidade, ou interesse em saber coisas da sua saúde, pois as possibilidades hoje na Internet não são tão difíceis quanto antes. Se tiver esse interesse... os idosos conseguem.* (30-F-Acompanhada, 70). *[...] tenho oportunidade de exercitar minha mente, e envelhecer melhor* (32-F-Acompanhada, 60). 

 A fragmentação sob a ótica de dois mundos, o real e o virtual, é complementada. Embora a luta contra o etarismo seja um desafio da década, as próprias pessoas idosas carregam estigmas ligados à menção de sentirem uma forma de pertencimento atrelada a duas realidades, referindo-se à primeira como uma realidade mais jovial e aceita socialmente pela chance de participar desse contexto tecnológico, e a segunda, um segmento de pessoas aposentadas, porém inseridas e engajadas nas oportunidades oferecidas pela tecnologia. *[...] quanto à tecnologia eu só falo que está sendo ótima para mim, envelhecer no mundo do modernismo é uma grande oportunidade para a nossa geração* (22-F-Acompanhada, 63). *Usar a Internet e o celular nesse mundo cheio de tecnologia, é importantíssimo para mim, porque eu vejo várias oportunidades de crescer por esse meio* (37-M-Acompanhado, 73). *[…] não conseguia ficar em casa quando me aposentei, foi de repente, e o celular me ajudou muito, ficava lendo sobre vários assuntos e isso me ajudou ser quem sou hoje, mais jovial, pois depois dos 60 é complicado, é difícil olhar no espelho e ver que chegou numa idade em que as pessoas não te aceitam em todos os lugares com a mesma atenção de antes* (20-F-Sozinha, 60). 

 A sensação de bem-estar é percebida diante da satisfação de entender o envelhecimento como um processo irreversível, porém passível de ser construído de forma saudável cuidando da saúde de forma global. *Por mais que eu participe das atividades físicas no centro de convivência para me preparar para envelhecer bem, vai chegar um momento que vai ficar mais limitado. Me sinto bem usando o celular nesse tempo, sabendo que tenho capacidade de participar, aprender, tanto intelectualmente quanto fisicamente* (22-F-Acompanhada, 63). *Estive numa depressão profundíssima quase chegando assim às vias de fato, nesse sentido o grupo me ajudou muito com a parte social, começar conversar, ouvir, ler, vídeos de objetos antigos que dá para revisitar, parece que faz um resgate da nossa alma, vai lá no fundo do poço e puxa a gente e fala… ó tudo isso você tem de história na tua vida, foi muito agradável e tem sido muito bom! mudou a minha vida* (20-F-Sozinha, 60). 

 Por outro lado, sentem-se preocupados em relação a situações específicas, como golpes no ambiente virtual que são comuns e que acometem, principalmente, as pessoas idosas. O medo de exposição excessiva em redes sociais virtuais é um sentimento que acompanha as pessoas idosas conectadas na Internet em virtude da publicização de dados pessoais. *[…] a pessoa não sabe que aquela mensagem é maldosa e passa para gen* te [...] *tem também a pessoa que tem segunda intenções, ao passar para um terceiro, está cometendo um crime* (8-M-Acompanhado, 86). *Fico receoso e com pé atrás para coisas de dinheiro e banco, isso não faço nada* (12-M-Acompanhado, 86). *[...] não é falta de orientação (cair no golpe), mas é um minuto de bobeira e já foi* (22-F-Acompanhada, 63). *[...] tenho medo porque lá (em rede) a gente quase não tem privacidade, não coloco foto, não comento minhas idas e vindas, fico muito exposto. Nas redes sociais não participo, não gosto dessa exposição toda, prefiro mais discrição* (10-F-Acompanhada, 65). 

Ao temerem os perigos da Internet, eles reconhecem e atribuem as fragilidades à limitação dos conhecimentos tecnológicos, diferindo de jovens que já são nativos digitais e possuem aptidão nessa área inerente ao desenvolvimento humano.

 As pessoas idosas, mesmo com limitações, reconhecem que o novo modo de pensar, com o uso do *smartphone,* permite a participação e o engajamento no que eles denominam de “ritmo da tecnologia”. *[...] uma criança pega o celular e mexe em tudo, a gente tem mais dificuldade porque começou muito tarde* (1-F-Acompanhada, 70). *[...] o idoso tem muita importância na sociedade, apesar dos apesares, a gente vê tanto preconceito… mas ainda há um alvo, um olhar para esse público idoso que está fazendo a máquina girar também. Eu me sinto “no ritmo” fazendo parte do processo tecnológico… Antes, 50 anos, já era uma pessoa velha e tonta, servia para cuidar de neto, hoje não, é bem diferente e eu acho louvável, ótimo* (22-F-Acompanhada, 63). *Hoje, quando abri o documento que lemos (TCLE) cliquei na canetinha, e apareceu a assinatura que eu fiz, [...] me senti esperta, lembrei como era para fazer e já fiz na hora* (28-F-Sozinha, 67). *Sinto que estou acompanhando o ritmo da tecnologia porque aprendo muito e até ensino. Tem coisa que eu aprendi que meus netos não sabiam, como do microfone preto, aperte para falar. Eles não sabiam disso, e eu que ensinei para eles* (29-F-Sozinha, 69). 

## Discussão

 Este estudo compreendeu o que a pessoa idosa sente e reconhece ao utilizar o *smartphone* no seu cotidiano, assim como a forma com que tal experiência incide no processo de envelhecimento saudável. Difere-se de estudos anteriores que abordam a pessoa idosa como aprendiz de tecnologia e inova-se ao centralizá-la como protagonista, usuária dos recursos tecnológicos, e dotada de uma nova mentalidade em busca de competências digitais para aplicá-las em seu curso de vida. 

 Corroboramos os estudos anteriores ^(^ 9 ^,^ 35 ^)^ , ao apontarmos que as pessoas idosas, ao perceberem o *smartphone* como benefício na vida diária, utilizam-no cada vez mais em suas rotinas e isso se torna um fator determinante para a sua inclusão nas diversas atividades pessoais. No entanto, a disposição das pessoas idosas para utilizar as tecnologias varia de acordo com sua expectativa de proveito e com a crença de que, por meio delas, obter-se-á ajuda para manter-se seguros ^(^ 16 ^-^ 17 ^,^ 35 ^,^ 38 ^)^ . 

 Trazemos evidências que demonstram que as pessoas idosas se reconhecem mais independentes intelectualmente e mais autônomas em suas atividades diárias ao usarem *smartphone.* Nota-se, em outro estudo, que a maioria das teorias do envelhecimento não considera a variabilidade individual, social ou cultural dos objetivos e expectativas das pessoas idosas ^(^ 38 ^)^ . 

 No uso cotidiano ou na aplicabilidade dos avanços tecnológicos na vida da pessoa idosa, a independência é percebida como um fator importante para o envelhecimento bem-sucedido ^(^ 38 ^)^ . Dessa forma, apresentamos, num contexto individualizado, os reconhecimentos e os sentimentos da pessoa idosa engajada tecnologicamente e, a partir disso, considerando os nossos resultados, entendemos que para construção de uma vida significativa para as pessoas idosas é preciso pertencer, reconhecer, sentir. Assim, nossos resultados apontaram para a interação de vários sentimentos a partir de experiências com o uso da tecnologia do *smartphone* , e para a ocorrência cruzada entre o reconhecimento desses sentimentos e suas representações no desdobramento ao longo de suas experiências, de forma que tais fatores oportunizam o envelhecimento saudável, conforme propõe o diagrama de Sankey. 

 Em relação à utilidade percebida do ponto de aceitação de tecnologia proposto pelo modelo UTAUT ^(^ 16 ^)^ e teoria STAM ^(^ 17 ^)^ , a pessoa idosa precisa considerar a aplicabilidade da tecnologia e suas ferramentas no cotidiano. Claramente, observa-se, neste estudo, que mesmo que a pessoa idosa utilize funcionalmente o *smartphone,* de forma que isso a satisfaça, é preciso estar inserida na dinâmica do aprendizado contínuo. As experiências que alicerçam os sentimentos das pessoas idosas ao utilizarem os *smartphones* estão relacionadas à sua própria percepção de competência e confiança para manusear o equipamento ^(^ 22 ^)^ . 

 Na China, um estudo realizado com pessoas idosas sobre os motivos para o uso de *smartphones* apontou a comunicação e a busca de informação como influenciadoras na satisfação com a vida, sendo relacionadas com a alfabetização digital ^(^ 39 ^)^ , reafirmada pelos nossos resultados. 

 Por conseguinte, é importante compreender que os fatores sociais – tais como o apoio familiar e dos pares, assim como os padrões culturais – auxiliam na aquisição de competências digitais pelas pessoas idosas, assim, a sua utilização na vida cotidiana reflete em mudanças que promovem o envelhecimento saudável ^(^ 1 ^)^ . 

 Para além do apoio informal, profissionais das mais diversas áreas podem auxiliar as pessoas idosas, especialmente os profissionais de saúde. Superando as barreiras de acesso aos cuidados presenciais, eles podem recomendar conteúdos e aplicações que contenham informações de saúde ^(^ 25 ^)^ . No Reino Unido, mais da metade das pessoas idosas com 65 anos ou mais estão dispostas a utilizar recursos digitais para a saúde. Contudo, a vontade de utilizar aplicativos de saúde recomendados por profissionais diminui à medida que a idade avança ^(^ 12 ^)^ . Esses dados reforçam a urgência de tratar assuntos dessa temática com pessoas idosas mais velhas, entendendo suas necessidades. 

 Na China, discute-se a prioridade que deve ser dada em remover alguns obstáculos na oferta de ferramentas, abrangendo o *design* dos produtos, que se concentra exclusivamente nos jovens e suas prioridades, demonstrando o preconceito relacionado à idade e um ambiente externo que é anti-facilitador e indutivo. Em outras palavras, as tecnologias são direcionadas conforme a idade e infraestrutura, mas também devem ser repensadas de acordo com as necessidades do processo de envelhecimento ^(^ 40 ^)^ . 

 Algumas descobertas trazem que não há unanimidade sobre os benefícios ou malefícios apropriados pelas pessoas idosas por meio da adoção da prática recorrente do uso do *smartphone* , assim, há fusão das influências percebidas/reconhecidas ^(^ 3 ^)^ . Este estudo mostrou um sentimento de otimismo, refletido em sensações de bem-estar percebido, o que foi demonstrado no diagrama de Sankey em co-ocorrência a outros sentimentos. Outras pesquisas relatam questões positivas acerca das influências recebidas entre as pessoas idosas ao utilizarem o *smartphone* e na sua aplicação em suas vidas ^(^ 3 ^,^ 41 ^-^ 42 ^)^ . 

 Os diversos sentimentos experimentados pelas pessoas idosas em relação à oportunidade de estarem conectadas em rede com outras pessoas da mesma faixa etária e com o mundo digital geram experiências de participação digital social, de competência técnica, de melhora de função física, cognitiva ^(^ 22 ^)^ e de conexão com familiares, inclusive em situações de vulnerabilidade ^(^ 21 ^)^ . Nesse sentido, também trouxemos evidências de que pessoas idosas sentem-se privilegiadas frente aos pares que ainda não desenvolveram habilidades com ferramentas digitais, confirmando os resultados de outro estudo que apoia o treinamento em tecnologia como gerador de um impacto positivo no uso de *smartphones* quando se refere ao bem-estar ^(^ 35 ^)^ . 

 O fato de se sentir privilegiado não elimina a necessidade de examinar criticamente o modo de acesso dos demais que estão sob um pré-conceito de marginalização. Destaca-se os resultados de um recente estudo de revisão, abordando o preconceito relacionado à idade nos sistemas de inteligência artificial, percorrendo os caminhos do desenvolvimento tecnológico e reiterando a existência de uma segregação ^(^ 43 ^)^ . 

 O uso do *smartphone* promove uma aproximação que perpassa questões etárias, proporcionando às pessoas idosas a experiência de pertencimento ao mundo digital ao participarem ativamente da TIC ^(^ 38 ^)^ . 

Este estudo alinha-se ao marco conceitual da Década do Envelhecimento Saudável, o qual traz, como objeto de percepção e ação, a luta contra o etarismo. Segundo a Organização Pan-Americana de Saúde, o etarismo é uma discriminação por idade que resulta em uma saúde mais precária, no isolamento social, em mortes prematuras além de gerar ônus de bilhões aos cofres públicos em nível global.

 Evidenciamos pensamentos das próprias pessoas idosas participantes, que reconhecem alguns fatores que condicionam ou restringem a competência digital em relação aos jovens que são nativos desse tempo. No mundo todo, a exemplo de países asiáticos como o Japão, Singapura e Tailândia ^(^ 1 ^)^ , assim como na América do Norte ^(^ 15 ^)^ e América do Sul, como o Brasil ^(^ 14 ^)^ existem divergências em relação à adoção de tecnologia por meio do *smartphone* entre jovens e pessoas idosas, mesmo que tenha sido percebida uma diminuição do fosso existente entre as extremidades etárias com o passar das décadas. 

 Nosso estudo aponta que as pessoas idosas possuem sentimentos de integração e pertencimento alinhavados aos conceitos inovadores à medida que eles surgem e se desenvolvem. Assim, estigmas e embaraços trazidos pelas próprias pessoas contribuem para o envelhecimento ativo e bem-sucedido, incluindo o preconceito de idade em relação ao uso e *design* da tecnologia digital ^(^ 38 ^)^ . Nesse sentido, produtos desenvolvidos especialmente “para” e “por” pessoas idosas promovem a diminuição do preconceito de idade. 

 Medos ligados à insegurança, à incerteza e receios são comuns entre pessoas idosas que utilizam ferramentas digitais para comunicação. Como contribuição negativa, destacamos que golpes virtuais e o medo de exposição excessiva podem levar as pessoas idosas a se retraírem nas redes sociais virtuais, tornando-se um fator impeditivo para uma participação ativa ^(^ 42 ^,^ 44 ^)^ . 

 Há um sentimento de medo implicado no reconhecimento da presença num novo contexto de comunicação, com limitações naturais do processo de transição. Corroborando isso, algumas barreiras dificultam o uso da tecnologia do *smartphone* por pessoas idosas, incluindo o medo, a falta de conhecimento, a preocupação em relação à complexidade, habilidade física, e dificuldade de comunicação ^(^ 35 ^)^ . Ressalta-se que as aplicações virtuais não devem ser desenvolvidas especificamente para públicos específicos, como as pessoas idosas, pois isso resulta em facilidade de adaptação e aprendizagem, o que, consequentemente, implica na permanência de uso das ferramentas e dispositivos virtuais em médio e longo prazo ^(^ 9 ^,^ 45 ^)^ . 

 Os achados apontam para fragilidades que se interceptam à restrição de conhecimentos tecnológicos. Mesmo com essas restrições, percebeu-se sentimentos de engajamento tecnológico por parte das pessoas idosas, fortalecendo resultados de outros estudos ^(^ 35 ^,^ 38 ^)^ , que apontam que alguns fatores são facilitadores e outros dificultadores no uso de formas que diversifiquem o apoio institucional e familiar para orientação do desenvolvimento pessoal com novas tecnologias, além das motivações e experiências frente ao processo de envelhecimento ^(^ 38 ^)^ . 

 Estudamos a percepção e os sentimentos das pessoas idosas saudáveis, todavia, a literatura traz dados sobre associações do uso desregrado e excessivo de tecnologia, especialmente do *smartphone* por pessoas idosas, que impactam na qualidade do sono devido a fatores de estresse e ansiedade ^(^ 46 ^)^ . 

 De sobremaneira e intencionalmente, o desenvolvimento das competências digitais e a formação tecnológica para o público de pessoas idosas precisam originar-se das intenções e comportamentos de uso. Isso possibilitará o desenvolvimento de modelos de aceitação em estudos futuros ^(^ 35 ^)^ . 

 Identificamos limitações neste estudo relacionadas ao direcionamento de evidências apenas para pessoas idosas que já possuem contato com as TICs, incorporando *smartphones* em suas rotinas e, ainda, orientadas por profissionais para tal uso, em um projeto de extensão. Não foi considerada a intencionalidade daquelas pessoas idosas que ainda desejam integrar-se, mas que não tiveram oportunidade. Sugere-se explorar essa temática em novas investigações e perspectivas mais diversificadas em termos étnicos, numa abordagem baseada na comunidade, por exemplo, o que poderá levar a uma melhor compreensão do impacto das TICs no envelhecimento saudável. 

Tanto sob a ótica da inclusão social digital quanto sob a ótica da promoção da saúde, este artigo inova-se por trazer a percepção dos efeitos do uso da tecnologia e a forma como incidem na vida das pessoas idosas. Partindo de suas experiências, seu curso de vida e motivações pessoais fornecem subsídio para o desenvolvimento de estratégias que podem ser guiadas por profissionais de saúde e propostas para a comunidade e para o público de pessoas idosas. O alinhamento da busca por competências digitais ao cotidiano atua no combate ao etarismo e no envelhecimento saudável, a longo prazo, contribuindo com afinco para o avanço do conhecimento científico para a área de saúde e enfermagem.

## Conclusão

 A compreensão dos sentimentos e reconhecimentos de pessoas idosas em relação ao uso da tecnologia do *smartphone* no cotidiano nos faz vislumbrar que, por vezes, as pessoas idosas ainda vivem segregadas dos jovens no contexto da tecnologia. Mesmo sentindo-se integradas, reconhecendo uma nova mentalidade e oportunidade de otimização de independência ao utilizarem o *smartphone* , as experiências das pessoas idosas revelam uma fragmentação entre real e virtual. Contudo, esse fato não é determinante para que este processo seja estático. Gradualmente, por meio do reconhecimento de suas necessidades em relação à tecnologia, principalmente quanto às TICs, a percepção da utilidade e aplicabilidade no cotidiano, a identificação com pares, a busca por competências digitais tem tornado a pessoa idosa em protagonista da aprendizagem, reverberando no decorrer de seu envelhecimento. 

 A permanência das pessoas idosas no engajamento virtual notabiliza condições favoráveis para promoção de saúde no processo de envelhecimento. A posse do *smartphone* não garante seu uso, nem a inclusão social digital, mas estímulos profissionais em grupos virtuais e orientações sobre práticas saudáveis os integram em um ambiente de recursos e informações aplicáveis ao processo de envelhecimento saudável. 

Adicionalmente, a enfermagem tem condições de contribuir no processo de tomada de decisões no campo virtual, podendo se sobressair como liderança, conduzindo a estratégias que mudem a realidade conceitual e prática de etarismo.
